# Dimethyl sulfide protects against oxidative stress and extends lifespan via a methionine sulfoxide reductase A‐dependent catalytic mechanism

**DOI:** 10.1111/acel.12546

**Published:** 2016-10-28

**Authors:** Xin‐Lei Guan, Peng‐Fei Wu, Sheng Wang, Juan‐Juan Zhang, Zu‐Cheng Shen, Han Luo, Hao Chen, Li‐Hong Long, Jian‐Guo Chen, Fang Wang

**Affiliations:** ^1^ Department of PharmacologySchool of Basic MedicineTongji Medical CollegeHuazhong University of Science and TechnologyWuhan430030China; ^2^ Department of PharmacyWuhan Puai HospitalTongji Medical CollegeHuazhong University of Science and TechnologyWuhan430033China; ^3^ Key Laboratory of Neurological Diseases (HUST)Ministry of Education of ChinaWuhan430030China; ^4^ The Key Laboratory for Drug Target Researches and Pharmacodynamic Evaluation of Hubei ProvinceWuhan430030China; ^5^ Laboratory of Neuropsychiatric DiseasesThe Institute of Brain ResearchHuazhong University of Science and TechnologyWuhan430030China; ^6^ School of Life Science and TechnologyHuazhong University of Science and TechnologyWuhan430074China; ^7^ The Collaborative Innovation Center for Brain ScienceWuhan430030China

**Keywords:** cell death, dimethyl sulfide, homology model, lifespan, methionine sulfoxide reductase A, oxidative stress

## Abstract

Methionine (Met) sulfoxide reductase A (MsrA) is a key endogenous antioxidative enzyme with longevity benefits in animals. Only very few approaches have been reported to enhance MsrA function. Recent reports have indicated that the antioxidant capability of MsrA may involve a Met oxidase activity that facilities the reaction of Met with reactive oxygen species (ROS). Herein, we used a homology modeling approach to search the substrates for the oxidase activity of MsrA. We found that dimethyl sulfide (DMS), a main metabolite that produced by marine algae, emerged as a good substrate for MsrA‐catalytic antioxidation. MsrA bounds to DMS and promoted its antioxidant capacity via facilitating the reaction of DMS with ROS through a sulfonium intermediate at residues Cys72, Tyr103, and Glu115, followed by the release of dimethyl sulfoxide (DMSO). DMS reduced the antimycin A‐induced ROS generation in cultured PC12 cells and alleviated oxidative stress. Supplement of DMS exhibited cytoprotection and extended longevity in both *Caenorhabditis elegans* and *Drosophila*. MsrA knockdown abolished the cytoprotective effect and the longevity benefits of DMS. Furthermore, we found that the level of physiologic DMS was at the low micromolar range in different tissues of mammals and its level decreased after aging. This study opened a new window to elucidate the biological role of DMS and other low‐molecular sulfides in the cytoprotection and aging.

## Introduction

Reactive oxygen species (ROS), including superoxide ($O_{2}^{-\cdot }$), hydrogen peroxide (H_2_O_2_), and hydroxyl radical, is important cellular toxicants that generated as products or by‐products in cells. A series of antioxidant mechanisms maintain intracellular redox homeostasis. When they deficit, overproduction of ROS causes DNA mutations and protein dysfunction, which eventually leads to cell injury and aging. The thiomethyl group that exists on the surface exposed methionine (Met) residues has been recognized as a key endogenous antioxidant defense (Ruan *et al*., 2002; Davies, 2005; Wood *et al*., 2009; Kim, 2013), providing for efficient scavenging of oxidants. To maintain the effective content of Met (Ruan *et al*., 2002), methionine sulfoxide reductase A (MsrA) plays a central role. MsrA reduced the oxidate of Met (methionine sulfoxide, MetO) back to Met and increased resistance of organisms to oxidative stress (Weissbach *et al*., 2002; Koc *et al*., 2004). Interestingly, MsrA was previously reported as a regulator of lifespan to increase longevity in animals, including *Drosophila*, yeast, and mice (Ruan *et al*., 2002; Koc *et al*., 2004; Chung *et al*., 2010). Gene expression decreased for MsrA in senescent cells and aged animals (Gabbita *et al*., 1999; Oien *et al*., 2009), and this decline was associated with the accumulation of oxidized proteins during aging. MsrA deficiency has been involved in the pathophysiology of aging‐related diseases (Yermolaieva *et al*., 2004; Pal *et al*., 2007; Oien *et al*., 2008; Prentice *et al*., 2008). Thus, pharmacological enhancement of MsrA function seems to be reasonable therapeutic strategy against aging in clinics. Only very few approaches are available to enhance MsrA function, such as activators like fusaricidin (Cudic *et al*., 2016), substrates including L‐Met and S‐methyl‐L‐cysteine (Wassef *et al*., 2007; Wood *et al*., 2009), and upregulation of endogenous MsrA (Novoselov *et al*., 2010; Wu *et al*., 2013a).

Despite MsrA has been identified in numerous organisms, and its role in the reduction in MetO has been described well (Ruan *et al*., 2002; Weissbach *et al*., 2002), little is known about how MsrA reduces ROS to confer its antioxidant capability. Previously, the antioxidation property of MsrA is considered as the natural antioxidation activity of Met residues (Davies, 2005). Recent studies by our group and other laboratories have indicated that MsrA may exert antioxidant effect through other potential pathways (Lim *et al*., 2011, 2013; Wu *et al*., 2013a; Fan *et al*., 2015), such as facilitating the reaction of Met with ROS via a stereospecific Met oxidase activity (Lim *et al*., 2011, 2013). Although Met emerges as a good substrate for the peroxidase activity of MsrA, there are many difficulties in systematic application of methionine *in vivo*, for its rapid metabolism and high risk of acute coronary events (Virtanen *et al*., 2006). Here, we used a homology modeling tool to search the better substrates for the stereospecific methyl sulfide oxidase activity of MsrA. We found that dimethyl sulfide (DMS), a main metabolite that produced by marine algae or fermentative bacteria (Scarlata & Ebeler, 1999; Avsar *et al*., 2004), was a good substrate for MsrA‐catalytic antioxidation. We demonstrated that the reaction between DMS and free radicals was catalyzed by an MsrA‐dependent mechanism and this enzymatic process leads to the beneficial effects of DMS on cytoprotection and longevity.

## Results

### MsrA promotes the ROS‐scavenging capacity of DMS via formation of sulfonium intermediate at residues Cys72, Tyr103, and Glu115 in its pocket

Homology modeling was used to analyze structure interactions between potential substrates and MsrA (see detail in supplementary methods). In previous studies, it has been shown that in the first step of MsrA‐catalytic oxidation, a sulfenic acid residue on Cys72 of MsrA forms by ROS attacking (MsrA‐CYO). Thus, we analyzed the orientation of different MsrA substrates in the active site of MsrA‐CYO (Fig. S1A–F). We found DMS adopts a preferred orientation with the distance of O‐S 3.8 Å (Table S1). In the presence of DMS binding, the distances of donor and acceptor atoms (D‐H···A) between the O atom from CYO72, the H atom from Tyr103‐OH, and Glu115‐COOH were 3.6 and 1.6 Å, respectively. Another question is whether the preferred orientation of DMS in the active site formed easily. Thus, energy barrier was used to evaluate the difficulty of orientation. Steering molecular dynamics (SMD) can operate single molecules to move closer or apart just like the working mode of atomic force microscopy. For oxidative reaction catalyzed by MsrA, substrates including DMS were pulled 16 Å away from MsrA and then pulled to the active site of MsrA. Curves of works showed how much energy was required to pull substrates to the active site as close as possible (Fig. S2A,B). It was obvious that much less energy was required for pulling DMS than Met substrates, indicating that the formation of sulfonium intermediate on DMS occurs more easily. As shown in Fig. S2C, DMS fitted properly in the catalytic pocket of MsrA, which also indicated DMS was a dominant substrate of MsrA.

**Figure 1:**
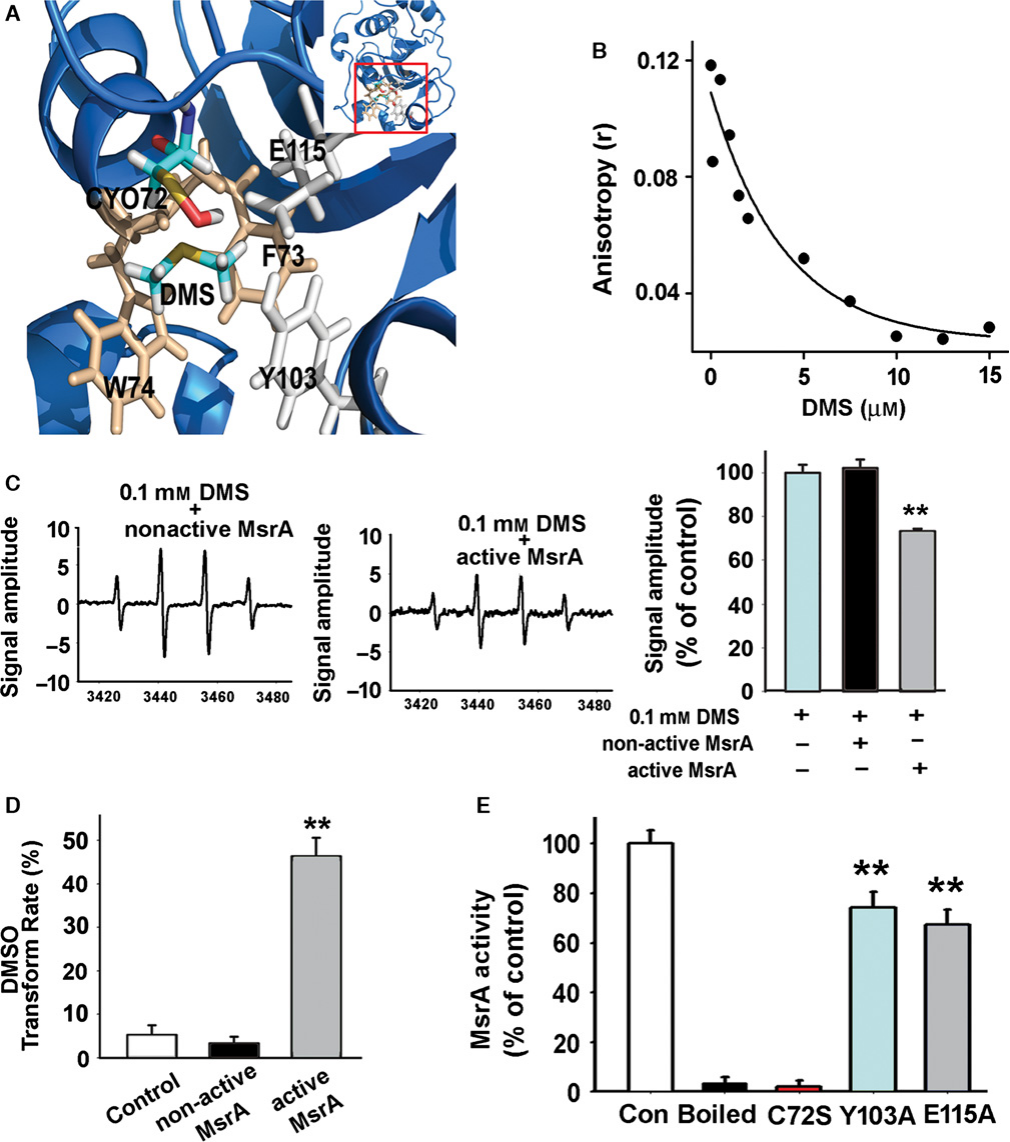
MsrA catalyzes the DMS‐mediated scavenging of radicals via formation of sulfonium intermediate at residues Cys72, Tyr103, Glu115. (A) DMS could easily bind to the catalytic pocket of MsrA, which is composed of side chains of Cys72, Phe73, Trp74, Tyr103, and Glu115. (B) Steady‐state fluorescence anisotropy values are shown as a function of increased concentrations of DMS. The concentration of MsrA was 3 μm. (C) The effect of 0.1 mm DMS on the signal intensity of DMPO‐OH adducts in the presence of MsrA or nonactive MsrA was evaluated under the same experimental conditions (*n* = 5, Student's *t*‐test, **P <* *0.01 vs. no enzyme group). The peak intensity of EPR was expressed as a relative change in comparison with 0.1 mm DMS treatment, which was set to 100%. (D) The transform rate of DMS into DMSO in the presence of active MsrA or nonactive MsrA was determined by GC‐MS method (*n* = 6, Student's *t*‐test, **P <* *0.01 vs. control). (E) The catalytic activity of single site mutant enzymes was detected by the reaction of scavenging OH˙ radicals. The activity of native enzyme was set as 100%, and boiled enzyme was set as negative control (*n* = 6, **P <* *0.01 vs. control, Student's *t*‐test). Data are expressed as mean ± SEM.

The DMS conformation from docking experiment indicated that DMS could easily bind to the catalytic pocket of MsrA (Fig. 1A), which was composed of side chains of Cys72, Phe73, Trp74, Tyr103, and Glu115. To confirm this point, firstly, we asked whether DMS could directly bind to the pocket of MsrA. The active rat recombined MsrA protein (3 μm) was incubated with different concentrations of DMS, and its binding was measured by fluorescence anisotropy. As shown in Fig. 1B, the anisotropy value of MsrA reduced with the increasing amount of DMS at near‐physiological concentrations (1–5 μm), indicating a direct binding of DMS to MsrA.

Previous studies indicate that DMS exhibits antioxidant function in marine algae (Sunda *et al*., 2002). We proposed that MsrA may increase the antioxidant activity of DMS. By luminol chemiluminescence assays in a Fenton reaction system, it was shown that H_2_O_2_‐induced luminescence was not inhibited by 0.1 mm DMS (97 ± 6% of control, shown in Fig. S3A). Neither MsrA nor nonactive MsrA alone (1 μm) had a perceptible effect on the luminescence intensity (Fig. S3B). Meanwhile, cotreated with 0.1 mm DMS and MsrA (1 μm), but not nonactive MsrA, remarkably reduced the luminescence signal intensity to 52 ± 5% (*P *<* *0.01, Fig. S3C). In electron paramagnetic resonance (EPR) assay, hydroxyl radical was induced by Fenton reaction and trapped by DMPO to form a stable spin adduct DMPO‐OH. The signal intensity of DMPO‐OH adducts was not changed by MsrA or nonactive MsrA alone (1 μm, Fig. S4). However, cotreatment with 0.1 mm DMS and MsrA (1 μm) decreased the DMPO‐OH signals to 73 ± 1% when compared to the group cotreated with 0.1 mm DMS and nonactive MsrA (Fig. 1C), which strongly suggested that MsrA increased DMS‐mediated reduction in OH˙. Then, we monitored the effect of MsrA on the reaction between ROS and DMS *in vitro* by detecting the reaction product. Increased amount of DMSO could be detected by GC‐MS, which supported by the catalytic activity of MsrA (Fig. 1D). MsrA facilitated the formation of DMSO from 3.3 ± 1.6% to 46.4 ± 4.3% (*P *<* *0.01).

As predicated, Tyr103 and Glu115 facilitated the transfer of oxygen atom to DMS via the formation of hydrogen bond (Fig. S2A). The Phe73 and Trp74 increased the binding of DMS to the pocket via the hydrophobic force. To validate this model, three single site mutants (Cys72Ser, Tyr103Ala, and Glu115Ala) were built. The peroxidase activity was measured by the ability to scavenge hydroxyl radicals in EPR tests (Fig. 1E). Notably, mutagenesis of Cys72 strongly diminished the peroxidase activity (Cys72Ser: 2 ± 2% of wild‐type), while the catalytic activity of Tyr103Ala and Glu115Ala decreased remarkably to 74 ± 6% and 67 ± 6% of wild‐type (*P *<* *0.01), respectively, indicating that Cys72 is the most critical one among these three residues. Furthermore, it was found that the predicted key residues (Cys72, Tyr103, and Glu115) were conservative in various organisms (Fig. S1), indicating that this catalytic mechanism may exist extensively.

### DMS alleviates oxidative stress and scavenges radicals in cell model

**Figure 2:**
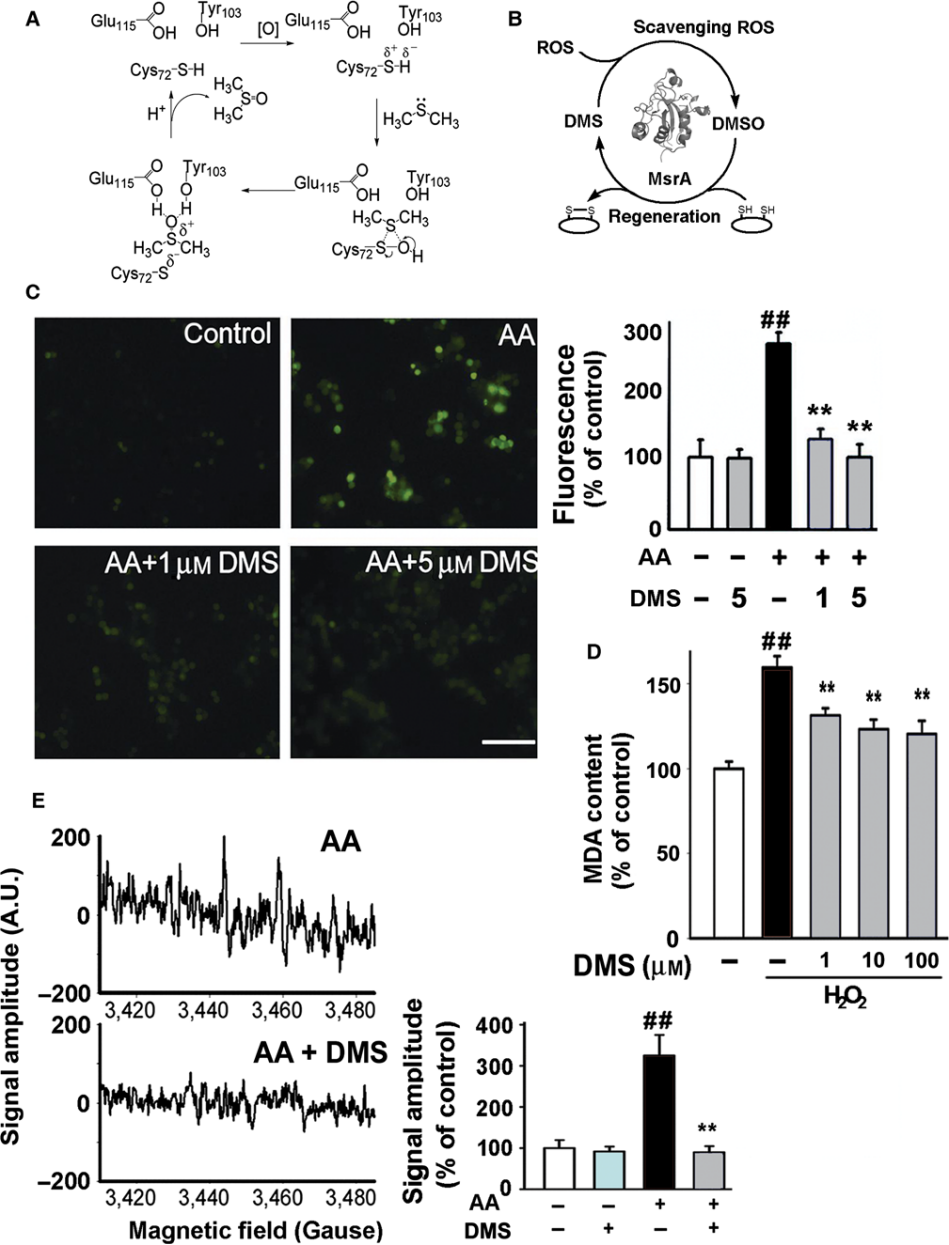
MsrA promotes the radical‐scavenging capacity of DMS. (A) We proposed the oxygen atom on the sulfenic acid was derived from H_2_O_2_ or other ROS. A lone pair of electrons on the DMS sulfur attacked the sulfenic sulfur to form the positively charged sulfonium intermediate. Glu115 and Tyr103 acted as a proton donor and stabilized by hydrogen bonding with DMS. With the release of DMSO, the reduced form of MsrA was formed. (B) The working hypothesis of DMS‐DMSO recycle system was illustrated. (C) PC12 cells were pretreated with 0, 1, or 5 μm DMS for 30 min and then incubated with AA (100 μg mL^−1^ for 30 min). The levels of intracellular ROS were detected with H_2_ DCFDA. The fluorescence intensity of DCF was determined (*n* = 6–9, one‐way ANOVA, ^##^ *P *<* *0.01 vs. control, **P <* *0.01 vs. AA.). The scale bar represented 100 μm. (D) Rat brain tissues were pretreated with DMS (0, 1, 10, or 100 μm for 30 min) and then incubated with H_2_O_2_ (250 μm for 30 min). The MDA contents were determined by a MDA test kit. *n* = 8, one‐way ANOVA, ^##^ *P *<* *0.01 vs. control, **P <* *0.01 vs. H_2_O_2_ group. (E) Cell culture medium with or without DMS (5 μM) was added into the culture wells for 30 min. And then, PC12 cells were incubated with AA (100 μg mL^−1^ for 10 min) in the presence of DMPO. Then, EPR signal of DMPO‐OH spin adduct was detected (*n* = 4, Student's *t*‐test, ^##^ *P *<* *0.01 vs. control, **P <* *0.01 vs. AA). The levels of EPR peak intensity were expressed as a relative change in comparison with the untreated control, which was set to 100%.

Based on the results of computation, we hypothesize that MsrA bounds to DMS and promoted its antioxidant capacity via facilitating the reaction of DMS with ROS through a sulfonium intermediate (Fig. 2A), followed by the release of dimethyl sulfoxide (DMSO) (Fig. 2B).

To further investigate the biological function of DMS, we observed the effect of DMS on cell lines. ROS production in PC12 cells was stimulated by antimycin A (AA), a mitochondrial respiratory complex III inhibitor. AA increased the levels of $O_{2}^{-\cdot }$, followed by conversion into other ROS, as judged by the fluorescence signals emitted by the oxidized forms of ROS probes. 2′,7′‐dichlorodihydrofluorescein (H2DCF) was used to evaluate total ROS. Application of AA (100 μg mL^−1^) for 0.5 h induced an increase of the total ROS level to 256 ± 15% of control (Fig. 2C, *P *<* *0.01). Pretreatment of PC12 cells with DMS (1 and 5 μm) significantly decreased the ROS level to 125 ± 14% and 100 ± 18% of control, respectively (*P *<* *0.01, Fig. 2C). We found that at both low concentration (1 μm) and high concentrations (10 and 100 μm), DMS significantly attenuated H_2_O_2_‐induced lipid peroxidation from 159 ± 7% to 131 ± 4%, 123 ± 6% and 120 ± 8% in the brain, respectively (*P *<* *0.01, Fig. 2D). Hydroxyl radical was detected by EPR assay. Cellular radicals were induced as previous report (Ohsawa *et al*., 2007). DMPO was added to capture the OH˙ radical. As shown in Fig. 2E, AA raised the spin signal to 325 ± 52% of basal level (*P *<* *0.01), and this was reduced to 90 ± 15% of basal level by 5 μm DMS (*P *<* *0.01).

### DMS has longevity benefits in *Caenorhabditis elegans* and *Drosophila melanogaster* via reducing accumulated oxidative damage

**Figure 3:**
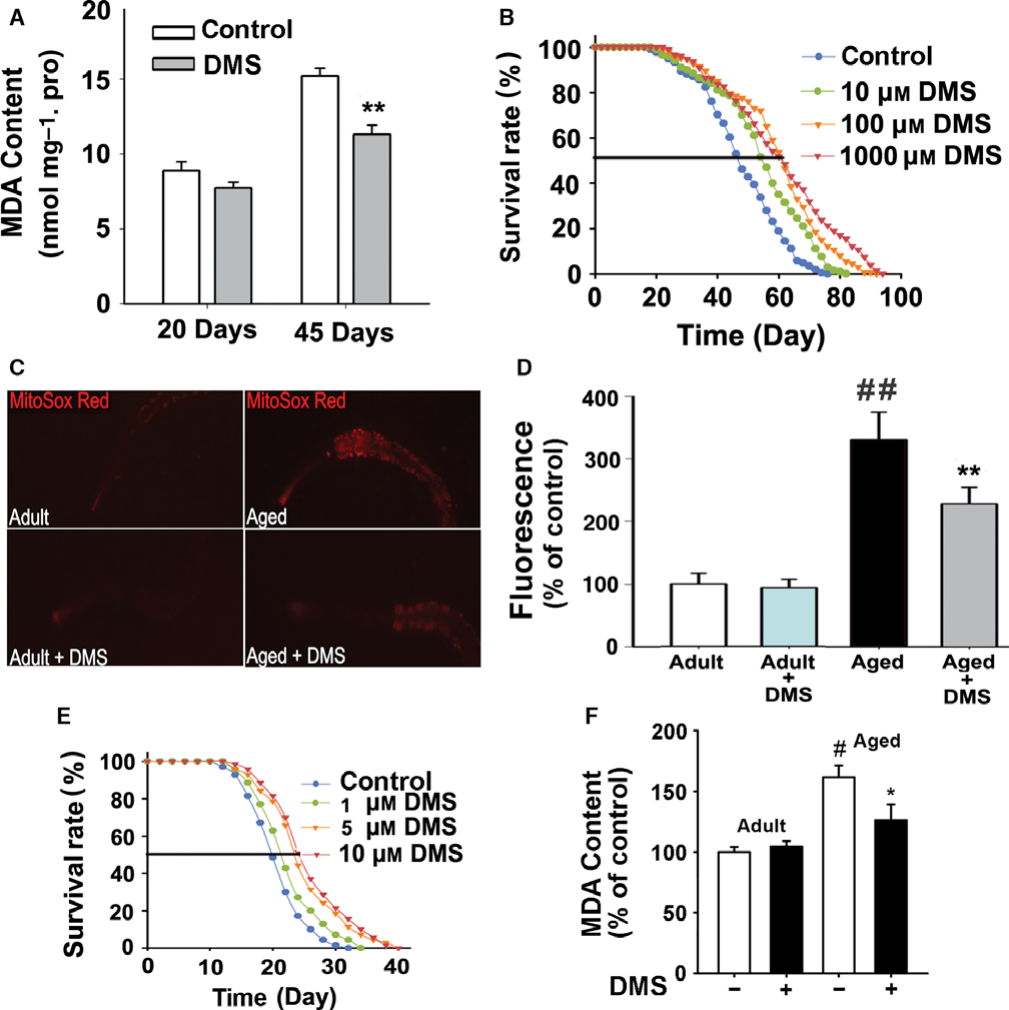
DMS has longevity benefits in *Caenorhabditis elegans* and *Drosophila* via reducing accumulated oxidative damage. (A) MDA contents in adult (20 days) and aged flies (45 days) were measured (20 flies in a sample, *n* = 6 independent samples, Student's *t*‐test, **P <* *0.01). (B) Lifespan analyses of wild‐type flies exposed to DMS (10, 100 or 1000 μm). *n* = 200, log‐rank *post hoc* Kaplan–Meier survival analysis, *P *<* *0.01 vs. control. (C) DMS reduced the levels of superoxide. Representative images of aged and adult worms labeled with the MitoSOX red. The scale bar represented 100 μm. (D) Quantification of superoxide levels by MitoSOX labeling (*n* = 6, Student's *t*‐test, ^##^ *P *<* *0.01 vs. adult, **P <* *0.01 vs. aged worms). (E) Lifespan analyses of wild‐type nematodes exposed to DMS (1, 5, or 10 μm). *n* = 70, log‐rank *post hoc* Kaplan–Meier survival analysis, *P *<* *0.01 vs. control. (F) MDA contents in adult (5 days) and aged worms (20 days) were measured (20 worms in a sample, *n *= 6 independent samples, Student's *t*‐test, ^#^ *P *<* *0.05 vs. adult control, *P <* *0.05 vs. aged control).

MsrA has longevity benefits in different organisms. As a substrate of the peroxidase activity of MsrA, DMS may exert longevity benefits. In *Drosophila*, the lipid oxidation product MDA decreased significantly with the treatment of DMS (10 μm) in aged *Drosophila* from 15.2 ± 0.5 nmol mg^−1^ protein to 11.3 ± 0.6 nmol mg^−1^ protein (Fig. 3A, *P *<* *0.01). We further tested whether DMS exerted lifespan‐extending properties in *Drosophila*. The mean survival time of control group was 48.25 ± 0.91 day. Meanwhile, the mean survival time was extended to 54.80 ± 1.06 day (10 μm), 59.95 ± 1.11 day (100 μm), and 55.99 ± 0.58 day (1000 μm) with DMS exposure (Fig. 3B, *P *<* *0.01). However, DMS did not prolong the lifespan of *Drosophila* at low concentrations (1, 2, or 5 μm, Fig. S5).

A similar effect of DMS was observed in *C. elegans*. MitoSOX red was used to detect the superoxide contents in aged *C. elegans* (Fig. 3C,D). The amount of superoxide in aged worms (reach median lifespan of control group) increased to 330 ± 44% of the superoxide level of adult worms (*P *<* *0.01). Treatment with 10 μm DMS remarkably decreased superoxide content to 228 ± 26% (*P *<* *0.01). Worms were fed with DMS medium at three different concentrations: 1, 5 and 10 μm. The mean survival time of DMS‐treated worms was extended from 21.00 ± 0.52 day to 22.77 ± 0.59 day (1 μm), 25.23 ± 0.72 day (5 μm), and 26.11 ± 0.71 day (10 μm), respectively (Fig. 3E, *P *<* *0.01). The MDA content decreased significantly with DMS (5 μm) treatment in aged worms from 161 ± 10% to 125 ± 13% of control (Fig. 3F, *P *<* *0.05). These results support the hypothesis that DMS mediates longevity via alleviating the accumulated oxidative damage in aged animals.

### DMS exerts cytoprotection against oxidative stress

**Figure 4:**
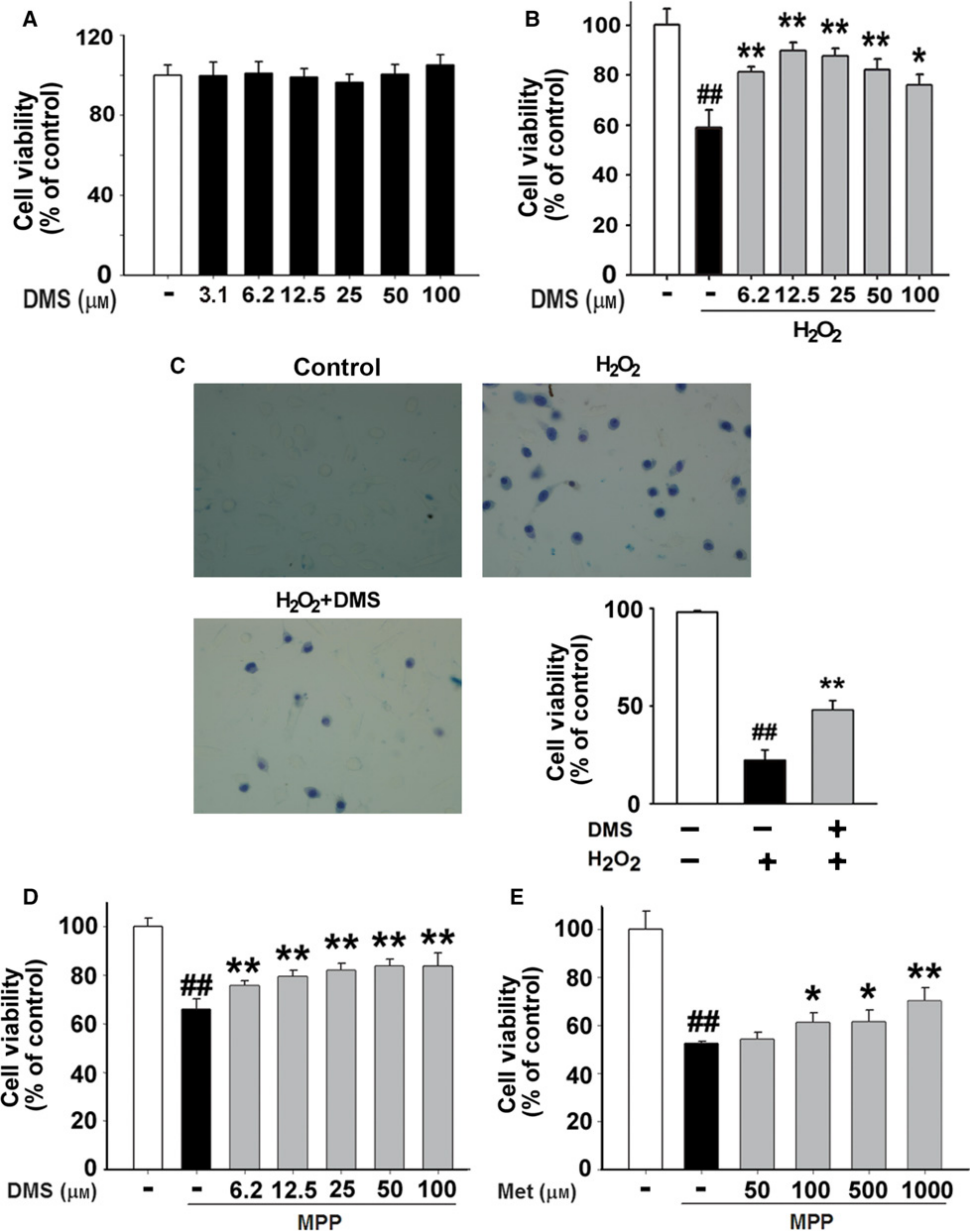
DMS exerts cytoprotection against oxidative stress. (A) Cell viability in SH‐SY5Y cells after treatment with DMS for 12 h (*n* = 8). (B) The SH‐SY5Y cells were pretreated with or without DMS (6.25, 12.5, 25, 50, or 100 μm) for 30 min. After the incubation of H_2_O_2_ (150 μm for 12 h), the cell survival rate was measured with MTT test kit (*n* = 8, ^##^ *P *<* *0.01 vs. control, *P <* *0.05, and **P <* *0.01 vs. H_2_O_2_ group). (C) The SH‐SY5Y cells were pretreated with or without 12.5 μm DMS. After 12‐h incubation with or without 150 μm H_2_O_2_, cell viability was tested by trypan blue test (*n* = 7, ^##^ *P *<* *0.01 vs. control and *^*^ *P *<* *0.01 vs. H_2_O_2_ group). (D‐E) The SH‐SY5Y cells were pretreated with DMS (6.25, 12.5, 25, 50, or 100 μm) or Met (50, 100, 500, or 1000 μm) for 30 min. After the incubation of MPP (2 mm for 48 h), the cell survival rate was measured with MTT test kit (*n* = 8, ^##^ *P *<* *0.01 vs. control, *P <* *0.05 and **P <* *0.01 vs. MPP group).

As a human original cell line, SH‐SY5Y cell was used to confirm the cytoprotective effects of DMS. As shown in Fig. 4A, we found that DMS did not change the cell survival rate of SH‐SHY5Y cell in micromolar range. In Fig. 4B, H_2_O_2_ (150 μm) was used to induce oxidative stress on SH‐SY5Y cells. After treatment for 12 h, the cell viability of SH‐SY5Y cells decreased to 59 ± 7% of control group. DMS increased the cell viability of SH‐SY5Y cells under oxidative stress to 81 ± 2% at 6.25 μm, 90 ± 3% at 12.5 μm, 88 ± 3% at 25 μm, 82 ± 4% at 50 μm, and 76 ± 4% at 100 μm (Fig. 4B). In the trypan blue test, DMS at 12.5 μm increased the cell viability of SH‐SY5Y cells under oxidative stress from 22 ± 5% to 48 ± 5% (Fig. 4C).

To test the effect of DMS on the concentration of extracellular oxidants, the MPP model was used to identify the cytoprotection of DMS. SH‐SY5Y cells were treated with or without 2 mm MPP for 48 h; then, the cell viability was evaluated by MTT test. Compared with MPP‐treated group (66 ± 4% of control, *P *<* *0.01 vs. control), the cell viability in DMS‐treated groups increased to 76 ± 2% (6.2 μm), 79 ± 3% (12.5 μm), 82 ± 3% (25 μm), 84 ± 3% (50 μm), and 84 ± 5% (100 μm, Fig. 4D). Meanwhile, the cell viability of Met‐treated group raised from 52 ± 1% to 61 ± 4% (100 μm), 62 ± 5% (500 μm), and 70 ± 6% (1000 μm) of control (Fig. 4E).

### MsrA knockdown abolishes the cytoprotective effects and longevity benefits of DMS

**Figure 5:**
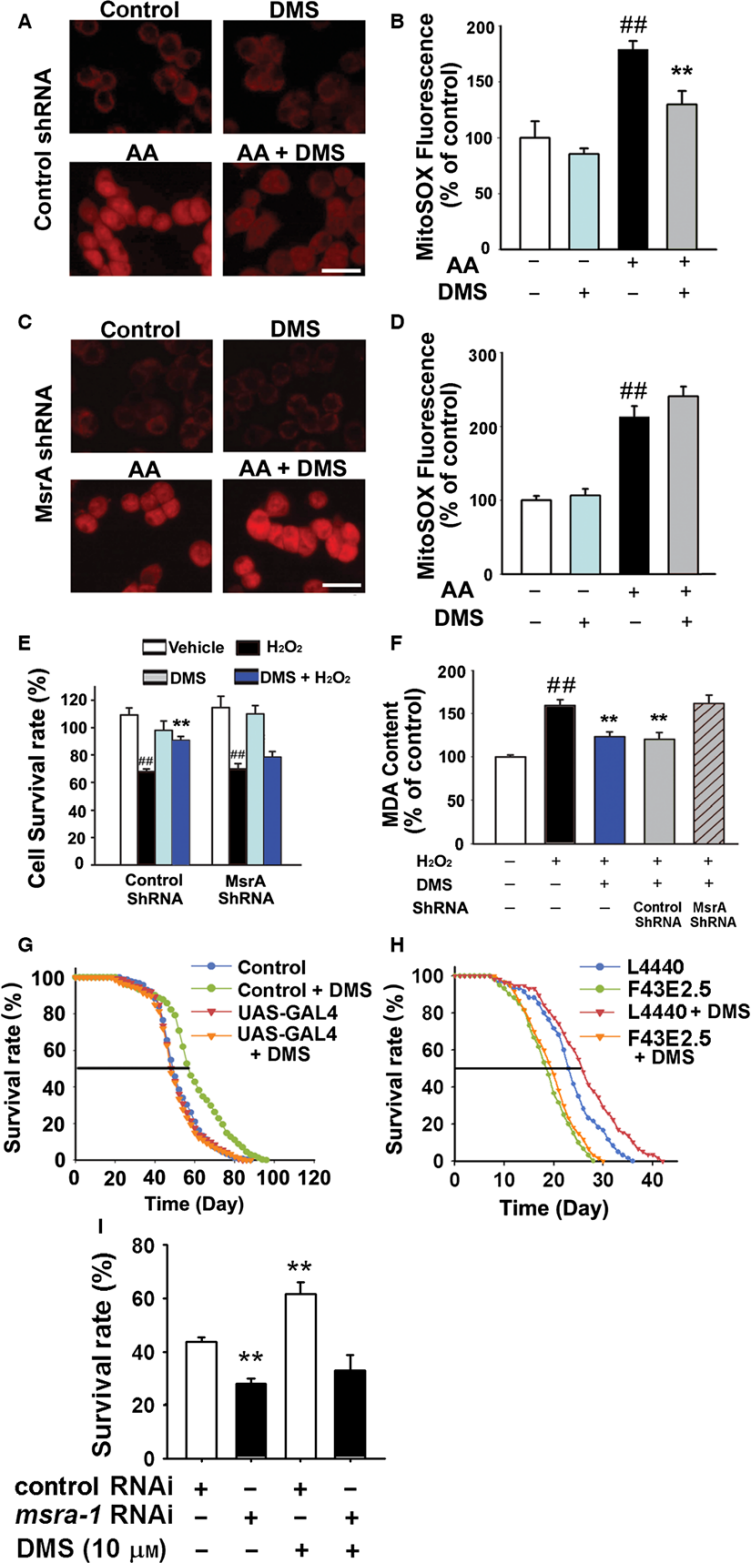
MsrA knockdown abolishes the cytoprotective effect and longevity benefits of DMS. PC12 cells were transfected with control shRNA (A) or MsrA shRNA (C). The transfected cells were pretreated with or without DMS (5 μm for 30 min) and then incubated with AA (100 μg mL^−1^ for 30 min). (B, D) The release of superoxide was determined by the MitoSOX red fluorescence. *n* = 6, Student's *t*‐test, ^##^ *P *<* *0.01 vs. control, **P <* *0.01 vs. AA. The scale bar represents 50 μm. (E‐F) The H_2_O_2_ (250 μm, 12 h) was introduced into the culture medium after treatment with DMS (5 μm for 30 min). (E) The cell survival rates were measured with MTT test (*n* = 8, Student's *t*‐test, ^##^ *P *<* *0.01 vs. control, **P <* *0.01 vs. H_2_O_2_). (F) MDA contents in cultured cells were measured (*n* = 8, Student's *t*‐test, ^##^ *P *<* *0.01 vs. control, **P <* *0.01 vs. H_2_O_2_). (G) Lifespan analyses of control flies and *dMsrA* RNAi flies (*n* = 400, log‐rank *post hoc* Kaplan–Meier survival analysis, *P *>* *0.05 vs. UAS‐GAL4). (H) Lifespan analyses of control worms (L4440) and F43E2.5 (*msra‐1* RNAi) worms (*n* = 60, log‐rank *post hoc* Kaplan–Meier survival analysis, *P *>* *0.05 vs. L4440). (I) Survival rates of worms (5‐day‐old adult) in each group were evaluated after 3 h in S‐basal buffer containing H_2_O_2_ (10 mm) in 20 °C (*n* = 60 from three independent tests, Student's *t*‐test, **P < 0.01 vs. control RNAi).

We then analyzed how DMS at low micromolar range scavenged ROS in PC12 cells. Considering the intrinsic catalytic function of MsrA in the oxidation of thiomethyl groups, we tested whether the antioxidation effect of DMS is dependent on MsrA. After treatment with lentiviral‐expressed specific short hairpin RNAs (shRNA) against MsrA (Fig. S6), the expression level of MsrA decreased to 22 ± 6% (*n* = 6, *P *<* *0.01 vs. control). As shown in Fig. 5A,B, DMS decreased the AA‐induced superoxide production in PC12 cells from 178 ± 8% of control group to 129 ± 8% (*P *<* *0.01). However, in MsrA shRNA group, the effect of DMS (10 μm) on AA‐induced superoxide production was abolished (Fig. 5C,D, 241 ± 13% in DMS group vs. 212 ± 14% in AA group of MsrA shRNA), suggesting that MsrA‐catalytic oxidation of thiomethyl group is involved in DMS‐mediated antioxidation. After MsrA knockdown by shRNA, the protective effect of DMS (5 μm) on cell viability against H_2_O_2_ was also abolished. With control shRNA, the DMS increased the cell viability of PC12 cells from 68 ± 2% in H_2_O_2_ group to 93 ± 3% in DMS group (Fig. 5E, *P *<* *0.01). With MsrA shRNA, DMS showed little effects on the cell viability under oxidative stress (70 ± 4% in H_2_O_2_ model group vs. 78 ± 4% in DMS group). The protective effect of DMS on lipid peroxidation was also abolished, which resembled the findings in the H_2_O_2_‐induced cell death. As shown in Fig. 5F, the MDA content of DMS group (162 ± 9%) showed no difference with that in H_2_O_2_ model group (160 ± 7%).

To confirm that MsrA involved in the mechanism of DMS‐mediated longevity, the level of MsrA was downregulated by UAS‐GAL4 system (Fig. S7A). As shown in Fig. 5G, DMS failed to prolong the lifespan in MsrA RNAi group, indicating that the lifespan extension effect of DMS is dependent on MsrA activity. Then, we used feeding RNAi (F43E2.5 plasmid) to decrease the *msra‐1* expression to 10 ± 5% of control group (L4440 plasmid) in *C. elegans* (Fig. S7B). In *msra‐1* RNAi group, lifespan was significantly shortened from 22.5 ± 0.8 day to 17.8 ± 0.6 day, which suggested that MsrA plays a critical role. DMS (10 μm) could not extend lifespan in *msra‐1* RNAi group (Fig. 5H). Thus, MsrA was required in the effect of DMS on worm longevity.

In Fig. 5I, H_2_O_2_ was used to introduce oxidative stress to test the resistance of worms. The survival rates of worms in each group were evaluated after 3 h in S‐basal buffer containing H_2_O_2_ (10 mm). The survival rate of DMS‐treated raised from 44 ± 2% (control RNAi) to 62 ± 4% (control RNAi with DMS, *P *<* *0.01). However, DMS failed to elevate the survival rate of worms treated with RNAi plasmid. The survival rate of *msra‐1* RNAi group (28 ± 2%) showed no significant difference with that of *msra‐1* RNAi with DMS group (33 ± 6%).

### Identification of DMS as an endogenous antioxidant that declines after aging

**Figure 6:**
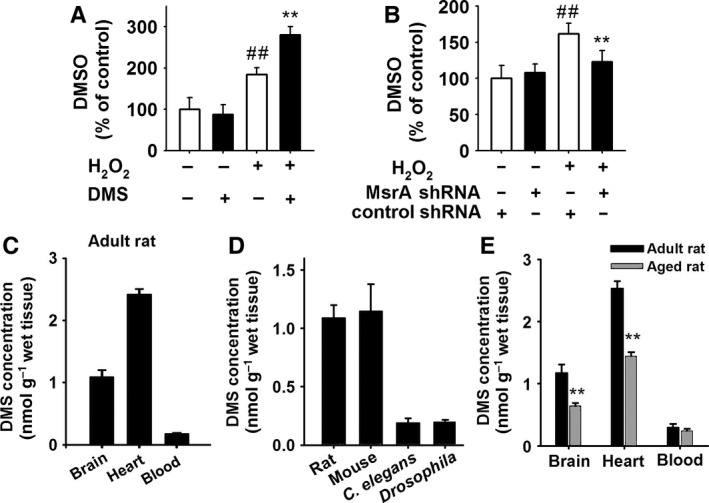
Identification of DMS as an endogenous antioxidant that declines with aging. (A) The levels of DMSO in PC12 cells were measured by HPLC after treatment of H_2_O_2_ (250 μm)/DMS (1 mm) for 12 h (*n* = 6, Student's *t*‐test, ^##^ *P *<* *0.01 vs. control, **P <* *0.01 vs. H_2_O_2_). (B) PC12 cells were transfected with MsrA shRNA or control shRNA before the treatment of DMS (1 mm). And then, the DMSO contents were evaluate after H_2_O_2_ (250 μm) treatment for 12 h (*n* = 6, Student's *t*‐test, ^##^ *P *<* *0.01 vs. control, **P <* *0.01 vs. H_2_O_2_). (C) The levels of DMS in different tissues of adult rat (brain, heart, and blood) were detected by GC‐MS assay, which were shown in a histogram (*n* = 6). (D)The tissues of different species (rat brain, mouse brain, and whole body of *Drosophila* or *Caenorhabditis elegans*) were also detected (*n* = 6). (E) The DMS levels in adult and aged rat samples were measured by SPME‐GC‐MS method (*n* = 6, **P <* *0.01 vs. adult, Student's *t*‐test).

We used HPLC to test the concentration of endogenous DMSO in PC12 cells (see details in supplementary methods). The endogenous cellular DMSO was at 5.73 nmol per 5 × 10^6^ cell count. As shown in Fig. 6A, the DMSO content in H_2_O_2_‐treated groups raised significantly to 184 ± 16% and 281 ± 20% of control, respectively (**P <* *0.01 vs. control, Fig. 6A). Then, MsrA shRNA was used to test whether MsrA played a vital role in the formation of DMSO (Fig. 6B). Knockdown of MsrA exhibited little effect on the concentration of endogenous DMSO in PC12 cells (Fig. 6B), which may be resulted from a bidirectional role of MsrA in the endogenous DMSO content: increasing DMSO generation under oxidative stress and reducing DMSO back to DMS using reducing substrates. Under H_2_O_2_‐induced oxidative stress, the DMSO formation in MsrA‐treated group decreased from 162 ± 14% to 123 ± 16% (*P *<* *0.01 vs. control shRNA with H_2_O_2_ group, Fig. 6B). We also tested the concentration of endogenous DMSO in blood and brain samples using both GC‐MS and HPLC. However, little endogenous DMSO was detected, which may be resulted by the limit of detection.

Previous reports have reported the existence of DMS in blood circulation of mammals (Blom *et al*., 1988; Miekisch *et al*., 2001). We firstly quantified the amount of endogenous DMS in different organisms by gas chromatography (GC)–mass spectrometry (MS). After being concentrated by solid‐phase microextraction (SPME), DMS was separated by GC followed by identification with MS (Fig. S8). The levels of DMS were 1.14 ± 0.24 nmol g^−1^ wet tissue (at about 1 μm) in rat brain, 2.42 ± 0.09 nmol g^−1^ wet tissue (at about 2 μm) in rat heart, and 1.09 ± 0.11 nmol g^−1^ wet tissue (at about 1 μm) in mice brain (Fig. 6A), respectively. The level of DMS in *C. elegans* and *Drosophila* was 0.19 ± 0.04 nmol g^−1^ wet tissue and 0.19 ± 0.02 nmol g^−1^ wet tissue, respectively (Fig. 6B), which was much lower in the tissues of mice and rats. More interesting, in aged group (20 months), the concentrations of DMS in brain and heart tissues of rats were significantly decreased from 1.17 ± 0.14 nmol g^−1^ to 0.64 ± 0.04 nmol g^−1^ (brain cortex) and from 2.53 ± 0.12 to 1.44 ± 0.07 nmol g^−1^ (heart), respectively (*P *<* *0.01, Fig. 6C).

## Discussion

The present study provided the first systematic identification of DMS as a catalytic antioxidant to protect cells and extend lifespan via the peroxidase activity of MsrA. After binding to the catalytic pocket of MsrA and forming of a positively charged sulfonium intermediate, DMS scavenged harmful radicals, reduced oxidative stress in the cell model, and mediated longevity. Considering the existence of DMS in the organisms is conserved, including *C. elegans*,* Drosophila*, mouse, rat, and human, DMS may act as a new member in the family of the physiologic antioxidative molecules.

DMS scavenges ROS via its thiomethyl groups. The antioxidation of DMS may not only be due to its natural antioxidant activity. Recent discovery of a MsrA‐centered catalytic mechanism raises the possibility of the physiologically regulated oxidation of thiomethyl groups in organism (Wu *et al*., 2013a; Fan *et al*., 2015). Thus, the antioxidation effect of DMS after MsrA knockdown was observed. We found that MsrA activity was required for the beneficial effects of DMS against oxidative stress. Furthermore, we established a computational model to visualize structural interactions between DMS and MsrA. Under oxidative stress, ROS induced a sulfenic acid residue on Cys72 of MsrA, followed by the reaction between oxygen atom on CYO72 and S atom of DMS. The Phe73 and Trp74 in MsrA facilitated DMS to bind the active center via hydrophobic bond, and Tyr103 and Glu115 increased the oxygen atom transfer from the CYO72 to the S atom of DMS via hydrogen bond. The peroxidase activity of MsrA significantly facilitated the reactivity of DMS at a low concentration. Furthermore, MsrA may increase the antioxidation of DMS via other mechanisms. Under oxidative condition, the oxydation product of DMS, DMSO, has been historically recognized as a strong scavenger of free radicals, which may secondarily react with radicals. The reaction between DMSO and OH˙ may underlie the total radical‐scavenging capacity of DMS. Under reduced condition, MsrA can reduce DMSO back into DMS, which may be critical in the availability of DMS. The mechanisms of DMS‐mediated radical‐scavenging capacity were illustrated in Fig. 2A,B.

In the present study, we have addressed novel MsrA‐catalytic antioxidant defense. We found that low‐molecular sulfides, such as DMS, could serve as the substrate of MsrA to scavenge oxidative stressors. The lone pair electrons on the methylthiol‐sulfur nucleophilicity of sulfide attack the sulfenic sulfur in active site to form the positively charged sulfonium intermediate. Then, MsrA catalyzes the transition and releases the sulfoxide. It should be noted that DMS and other thiomethyl‐containing compounds, such as free methionine, methyl mercaptan, and dimethyl disulfide, may work synergistically with the Met residues. An MsrA‐regulated endogenous catalytic antioxidant network of low‐molecular methyl sulfides may also confer a good antioxidation defense *in vivo*.

MsrA deficiency has been involved in the pathophysiology of aging‐related diseases (Yermolaieva *et al*., 2004; Pal *et al*., 2007; Oien *et al*., 2008; Prentice *et al*., 2008). Previous study has reported that msra‐1 absence decreases median survival of long‐lived daf‐2 worms (Minniti *et al*., 2009). In our study, lifespan in *msra‐1* RNAi group was significantly shortened from 22.5 ± 0.8 day to 17.8 ± 0.6 day, indicating that MsrA plays a critical role in lifespan of worms (Fig. 5H). In flies, reducing MsrA activity exhibited little effect on the lifespan. It seems that the role of MsrA in lifespan is conditionally important in response to certain stresses. As a catalytic antioxidant, under MsrA deficiency, the organism may be vulnerable to stress and other compensatory mechanisms may be activated to alleviate its effect on lifespan. Enhancement of MsrA system may be beneficial for lifespan by reducing accumulated oxidative damage, as previous reports have revealed that upregulation of MsrA system increases longevity in animals, including *Drosophila* and yeast (Ruan *et al*., 2002; Koc *et al*., 2004; Chung *et al*., 2010). Only very few approaches are available to enhance MsrA function, such as activators like fusaricidin (Cudic *et al*., 2016), supplement of its substrates (Wassef *et al*., 2007; Wood *et al*., 2009), and upregulation of endogenous MsrA (Novoselov *et al*., 2010; Wu *et al*., 2013a).One remarkable finding of the present study is that food supplement of DMS extended the lifespan of *C. elegans* and *Drosophila* via an MsrA‐dependent manner and improves the life condition under oxidative stress. These results support the connection between our observed effects on longevity and antioxidant‐mediated cytoprotective mechanisms. However, other multiple mechanisms may be involved in the effect of DMS on the ROS profile, such as alterations in metabolism and gene expression. Considering the low‐molecular weight, endogenous origin, and easy preparation, it is possible that supplement of DMS‐rich and methyl sulfide‐rich foods including beer, cheese, and marine products may serve as a potential nutritional strategy to enhance MsrA function and prevent aging.

As the major sulfide emitted to the atmosphere by the marine microorganisms (Wolfe *et al*., 1999; Todd *et al*., 2009), DMS has been historically recognized in the atmospheric circulation (Cameron‐Smith *et al*., 2011). Although current studies have focused on its role in the lower organisms (Wolfe *et al*., 1999; Sunda *et al*., 2002; Todd *et al*., 2007), DMS has been found in the blood circulation of mammals, including human being (DeBose & Nevitt, 2008; Mochalski *et al*., 2014), and recognized as a potent chemoattractant for animals and a disgusting component of bad breath (DeBose & Nevitt, 2008; Nevitt, 2008). However, other physiological role of DMS in mammals remains to be unmasked. We found that at near‐physiological level, DMS reduced the level of ROS in cell lines and alleviated oxidative stress. Thus, DMS may emerge as a physiologic cytoprotectant against oxidative stress‐induced cell death and aging.

In summary, this study shows that DMS induced cytoprotection against oxidative stress‐induced cell death and aging via an MsrA‐catalytic mechanism. A missing role of thiomethyl‐containing compounds, such as DMS, methyl mercaptan, and dimethyl disulfide, in the anti‐aging should been assigned in the mammals. With respect to analogues of DMS, its reaction priority and evolutionarily conservation suggest a similar function of DMS in humans.

## Material and methods

### Reagents

AA, dithiothreitol (DTT), H2DCF, H_2_O_2_, DMPO, DMS, DMSO, and penicillin–streptomycin were purchased from Sigma‐Aldrich (St. Louis, MO, USA). MitoSox red was purchased from Invitrogen (Waltham MA, USA). DMEM/F12, RPMI1640 and fetal bovine serum were purchased from HyClone (Logan, UT, USA). NaBH_4_ was obtained from Sinopharm chemical reagent (Shanghai, China). SPME holder and 75‐μm carboxen‐PDMS SPME fiber was purchased from Supelco (Bellefonte, PA, USA). Ni‐Trap nickel‐chelating column was obtained from Qiagen (Hilden, Germany). Thrombin kit was purchased from New England Biolabs (Ipswich, MA, USA). Fast Mutagenesis System was obtained from Transgene (Beijing, China). MDA and cell survival rate kits were obtained from Jiancheng Biotech (Nanjing, China).

### Animals

The wild‐type *C. elegans* N2 worms were grown on *E. coli* OP50 using standard techniques on 20 °C (Brenner, 1974). The RNAi worms were feed with *E. coli* HT115 containing plasmid L4440 or F43E2.5 (Minniti *et al*., 2009). The worms were kindly provided by Dr. An‐bing Shi. The wild‐type *Drosophila* stock w^1118^ was kindly provided by Dr. Jin Shan. Actin‐GAL4 and UAS‐dMsrA‐RNAi were procured from the Tsinghua Fly Center (THFC). The *Drosophila* strain w^1118^ was used in all control crosses and as the background for generation of transgenic lines. All *Drosophila* stocks were maintained, and all experiments were conducted at 25 °C on a 12‐h: 12‐h light: dark cycle at constant humidity using standard sugar/yeast/agar (SYA) media. Adult male Sprague Dawley rats and C57BL/6J mice were obtained from the Experimental Animals of Tongji Medical College, Huazhong University of Science and Technology. The rats/mice were housed on a controlled 12‐h: 12‐h light cycle at a constant temperature (24 ± 1 °C) with free access to water and food and allowed to acclimate a week. The use of animals for experimental procedures was conducted in accordance with the Guide for Care and Use of Laboratory Animals as adopted and promulgated by the National Institutes of Health. The experimental procedures were approved by the Animal Welfare Committee of Huazhong University of Science & Technology.

### DMS/DMSO assay

Different tissue samples were collected and prepared as described by published report (Al Mardini *et al*., 1984). In brief, the whole blood was collected in heparin anticoagulated tubes, sealed in glass GC bottles, and then stored at −80 °C before use. The samples was placed on ice, weighed, and added to four parts Tris–HCl buffer (pH 7.0) before sonication. One milliliter of aliquots was subsequently added to septum bottles and treated in the same way. The DMS produced was stripped, cryo‐trapped, redissolved in water, and then analyzed by SPME‐GC‐MS. After removal of DMS by purge and cryo‐trapping, DMSO was reduced to DMS using NaBH_4_ and analyzed by SPME‐GC‐MS. Headspace sampling of DMS was carried out with a SPME holder equipped with a 75‐μm carboxen‐PDMS SPME fiber. Samples were incubated for 5 min at 35 °C and then extracted for 10 min at the same temperature. Then, the GC/MS analysis was performed with GC‐MS analysis using Agilent 6890N‐5975B GC‐MS system (Agilent Technologies, Santa Clara, CA, USA). The injector temperature was 200 °C, and injections/SPME desorptions were made in splitless mode. Helium was used as a carrier gas with linear velocity 40 cm s^−1^. The MS analyses were carried out in a SIM scan mode, with a scan of *m/z* 47 and 62. Electron ionization was used at energy of 70 eV. The temperature of the ion source and the transfer line was 190 and 150 °C, respectively. The acquisition of chromatographic data was performed by means of the Chemstation Software (Agilent).

### Electron paramagnetic resonance (EPR)

Dimethyl pyridine N oxide (DMPO) was used as a free radical trapper. EPR signals were detected with a Bruker e‐scan EPR spectrometer (Burker, Karlsruhe, Germany) as previous report (Ohsawa *et al*., 2007). We produced hydroxyl radical (OH˙) by the Fenton reaction in the mixture of 0.25 mm H_2_O_2_ and 0.1 mm FeSO_4_ in the presence of 0.1 mm DMPO. The spin traps, DMS (0.1, 1, or 10 mm), and MsrA/nonactive MsrA (1 μm) were added before the Fe (II) and H_2_O_2_. Samples (20 μL) were loaded into a quartz tube, and the EPR spectra were recorded at room temperature. The EPR microwave power was set to 4.88 mW. The modulation frequency was 9.76 GHz. The time constant was 81.92 ms, and conversion time was 81.92 ms. A sweep time of 41.94 s was used. Each sample was scanned once. A sweep width of 100 G was used for experiments with DMPO. The receiver gain was set to 3.17 × 10^3^. Simulation and fitting of the EPR spectra were performed using the Bruker WinEPR program.

### Preparation of rat recombined MsrA

The rat recombined MsrA was prepared and purified from E. coli BL21, as described in our previous report (Wu *et al*., 2013b). In brief, MsrA coding region was ligated into the restricted pET‐32a (+) vector using phage T4 DNA ligase. BL21 cells were transformed with the recombinant plasmid and grown in LB medium. The cells were harvested, and cell lysis was applied to a 4 mL Ni‐Trap nickel‐chelating column. The N‐terminal His‐tag of recombinant peptide was removed using a Thrombin kit. The activity of recombinant MsrA was monitored by detecting both MetO‐reducing activity and methyl sulfoxide‐dependent oxidation of DTT.

### Construction of single site mutant plasmid of MsrA

The single site mutant plasmids were constructed with Fast Mutagenesis System. The primers were listed below as follows:

Cys72SerF:5′‐ TTTGGAATGGGCAGCTTCTGGGGAGCT ‐3′
R:5′‐ TGCCCATTCCAAATACAGCCATCTG ‐3′
Tyr103AlaF:5′‐ ACACGCAATCCCACCGCCAAAGAGGTC ‐3′
R:5′‐ GCGGTGGGATTGCGTGTGTAGCCTCCT ‐3′
Glu115AlaF:5′‐ AAAACCGGTCACGCAGCAGTCGTCCGG ‐3′
R:5′‐ GCTGCGTGACCGGTTTTTTCTGAGCA ‐3′

### Fluorescence anisotropy

The steady‐state fluorescence anisotropy measurements were performed with a PerkinElmer LS55 spectrofluorometer using a quartz cuvette at room temperature as described by previous report (Pillai *et al*., 2015). Excitation and emission slits with a band pass of 5 nm were used for all measurements. The anisotropy values were calculated from the equation below,

$$r\;=\;\frac{I_{\mathrm{VV}}-{\text{GI}}_{\mathrm{VH}}}{I_{\mathrm{VV}}+2{\text{GI}}_{\mathrm{VH}}}$$

where I_VV_ and I_VH_ are the measured fluorescence intensities with the excitation polarizer vertically oriented and emission polarizer vertically and horizontally oriented, respectively. G is the instrumental correction factor and is the ratio of the efficiencies of the detection system for vertically and horizontally polarized light and is equal to I_HV_/I_HH_.

### Cell culture

PC12 and SH‐SY5Y cell lines were obtained from JRDUN Biotechnology (Shanghai, China). The PC12 cells were cultured in RPMI1640 supplemented with 15% horse serum, 2.5% fetal bovine serum, and 100 U mL^−1^ penicillin–streptomycin. The SH‐SY5Y cells were cultured in DMEM/F12 supplemented with 10% fetal bovine serum and 100 U mL^−1^ penicillin–streptomycin.

### Construction of rat MsrA shRNA lentiviral expression vector

A third generation of self‐inactivating lentivirus vector (GeneChem, Shanghai, China) containing a CMV‐driven GFP reporter and a U6 promoter upstream of the cloning sites (Age I and EcoR I) was used for cloning shRNAs. The target sequence for rat MsrA was 5′‐AGCACGTCAGCTTTGAGGA‐3′ and for control scrambled shRNA was 5′‐TTCTCCGAACGTGTCACGT‐3′. PC12 cells were infected with lentivirus at a multiplicity of infection (MOI) of 20 for 8 h. Then, the medium was replaced with fresh complete medium. After 72 h, cells were observed under fluorescence microscopy to confirm that more than 80% of cells were GFP‐positive.

### MsrA homology modeling

See details in the Supplementary Methods section.

### Ligands affinity test

See details in the Supplementary Methods section.

### Intracellular Fenton reaction

See details in the Supplementary Methods section.

### Measurements of intracellular ROS

See details in the Supplementary Methods section.

### Lifespan experiments

See details in the Supplementary Methods section.

### Superoxide levels in *Caenorhabditis elegans*

See details in the Supplementary Methods section.

### mRNA isolation, reverse transcription, and qPCR

See details in the Supplementary Methods section.

### MDA and cell viability assay

See details in the Supplementary Methods section.

### HPLC assay method

See details in the Supplementary Methods section.

### H_2_O_2_‐induced oxidative stress

See details in the Supplementary Methods section.

### MsrA RNAi nematode lifespan assay

See details in the Supplementary Methods section.

### Statistical analysis

Data are expressed as mean ± SEM Significance of differences between groups was determined by Student's *t*‐test or *LSD* test *post hoc* ANOVA. The Kaplan–Meier method was used to compare the differences in survival rates between groups. A *P*‐value < 0.05 was considered statistically significant.

## Funding

This work was supported by grants from the National Basic Research Program of China (the 973 Program, No. 2013CB531303 to Dr. J.G.C.; No. 2014CB744601 to F.W.) and the National Natural Scientific Foundation of China (NSFC, No. 81302754 to P.F.W). It was also supported by PCSIRT (No. IRT13016), the National Key Scientific Instrument and Equipment Development Project of China (No. 2013YQ03092306) and Science Fund for Creative Research Groups of the Natural Science Foundation of Hubei Province (2015CFA020) to J.G.C.

## Conflict of interest

No competing financial interests exist.

## Supporting information


**Appendix S1** Methods.
**Fig. S1** DMS serves as a dominant substrate of MsrA.
**Fig. S2** The catalytic pocket model of DMS‐MsrA interaction.
**Fig. S3** DMS decreases the luminal chemiluminescence *in vitro*, while MsrA enhances the OH˙ scavenge property.
**Fig. S4** Neither MsrA nor non‐active MsrA alone has a perceptible effect on ESR signals.
**Fig. S5** DMS does not prolong the lifespan of *Drosophila* in low concentrations.
**Fig. S6** MsrA shRNA significantly inhibits the expression of MsrA in PC12 cells.
**Fig. S7** The effects of MsrA RNAi are evaluated by RT‐PCR and RT‐qPCR.
**Fig. S8** The detection method of DMS is built using GC‐MS.
**Table S1** Distances (Å) between atoms CYO‐S/CYO‐O and S atom; S from DMS, 1 EMS, MPS, L‐Met, AMN, Tripep, Hexpep.
