# Supplementary material for: Dimethyl sulfide protects against oxidative stress and extends lifespan via a methionine sulfoxide reductase A‐dependent catalytic mechanism
Source: Aging Cell. 2016 Oct 28;16(2):226–36. doi: 10.1111/acel.12546 (PMC5334523; doi:10.1111/acel.12546)
Supplement: Supplementary file 1 — Appendix S1 Methods. Fig. S1 DMS serves as a dominant substrate of MsrA. Fig. S2 The catalytic pocket model of DMS‐MsrA interaction. Fig. S3 DMS decreases the luminal chemiluminescence in vitro, while MsrA enhances the OH˙ scavenge property. Fig. S4 Neither MsrA nor non‐active MsrA alone has a perceptible effect on ESR signals. Fig. S5 DMS does not prolong the lifespan of Drosophila in low concentrations. Fig. S6 MsrA shRNA significantly inhibits the expression of MsrA in PC12 cells. Fig. S7 The effects of MsrA RNAi are evaluated by RT‐PCR and RT‐qPCR. Fig. S8 The detection method of DMS is built using GC‐MS. Table S1 Distances (Å) between atoms CYO‐S/CYO‐O and S atom; S from DMS, 1 EMS, MPS, L‐Met, AMN, Tripep, Hexpep. [file ACEL-16-226-s001.pdf]

## Supplementary Methods

### MsrA homology modeling

MODELLER suite (version 9.11)(Sali & Blundell 1993) was used to construct the model of rat MsrA, and the template was a known complex of methionine sulfoxide reductase A from Neisseria meningitides (PDB ID 3BQF) (Ranaivoson *et al.* 2008). Then residue CYS72 of MsrA are modified to its oxide state, just called CYO72 here, and the modified-MsrA was named as CYO72-MsrA for convenience. The coarse model of CYO72-MsrA was then refined by a 12 ns standard molecular dynamics in AMBER 12 (Case 2012b) until the ensemble attained its equillibium state to get the stable conformation of CYO72-MsrA. During the MD process, the CYO72-MsrA was solvated in a truncated octahedron box with 0.1M NaCl and several another $Na^+$ ions added to counter solute charges.

Ligands used in this study, including dimethyl sulfide(DMS), ethyl methyl sulfide(EMS), methyl propyl sulfide(MPS), L-Met, ACE-MET-NME were all prepared with academic maestro 9.6 from DESMOND molecular dynamics suite (Bowers *et al.* 2006) and all of them were minimized energetically for further studies.

According to the known crystal structure of MsrA (PDB ID 3BQF), all of ligands were docked manually to the active site of CYO72-MsrA to form various complexes, especially keeping the orientation of S-Me group being consistent with that of 3BQF structure. Again, all of complexes were refined in AMBER 12 with explicit solvent environment and the same ion strength above mentioned. After that, the ligands were pulled 16 angstroms away from the active site to obtain the starting structures for later Steered Moleculare Dynamics (SMD) simulations.

### Ligands affinity test

To disdinguish the difference of action between ligands and CYO72-MsrA, SMD method was conducted for those starting structures prepared above. First, those starting structures were solvated with similar conditions as before. One minimization was then done with restrained on solutes following one no-restraint minimization to get rid of steric clashes inside the ensembles. Next, all of those systems were heated

within 200 ps from 0K to 300K with weak restraints on solutes to prevent ligands shifting away from enzyme. After heating those systems completely, NVT simulations were arranged to all systems at 300K. All of NVT simulations were sustained a longer period of 10 ns with time step 2 fs and 5,000,000 steps altegher. Finally, several one-ns SMD simulations were preformed for every ligands, which pulled them into the active site of CYO72-MsrA from 16 angastroms to their own equillibrated states of complexes. In the mean time backward pulling simulations were done to pull ligands away from active site of CYO72-MsrA. The forward and backward works of pulling vs. a series distances can be figured altogether into one figure for later analysis.

All of MD simulations described here were completed in Amber 12 (Case 2012a) with CYO72-MsrA parameterized using the Amber 99SB-ildn force field (Lindorff-Larsen *et al.* 2010). Methionine sulfoxide residues of MsrA,CYO72, which represent the oxidized form of methionine, were parameterized with antechamber of Amber suites by using the GAFF force field and the charges of all non-standard residues including CYO and ligands DMS, EMS and MPS were calculated from a ORCA 2.8 quantum chemistry program package from the development team at the University of Bonn structure optimization were performed at the HF/6-31g(d) level of theory (Neese 2010). Spin-restricted Kohn-Sham determinants have been chosen to describe the closed-shell wave functions, employing the RI approximation and the tight SCF convergence criteria provided by ORCA. In addition, some calculations were conducted parallelly on GTX 690 GPU or 3.2G multi-core Xeon 5580 CPU to accelerate the processes of simulations.

**Intracellular Fenton reaction**

We preincubated PC12 cells with 100 μg/mlAA for 10 min in medium, washed once with phosphate-buffered saline (PBS, pH 7.2), and then exposed to the indicated concentration of DMS for 10 min in PBS(Zhang *et al.* 2007). As negative controls, AA and/or DMS were omitted.

## Measurements of intracellular ROS

The total intracellular ROS were determined by the 2´, 7´-dichlorodihydrofluorescein diacetate (H2DCFDA) assay. In brief, PC12 cells were seeded in a 6-well plate and loaded with H2DCFDA (20 μM, Sigma-Aldrich, St. Louis, USA) for 1 h followed by pretreatment with DMS (0.2, 1, and 5 μM) for 30min, and then treatment with antimycin A (100 ng/ml) (Sigma-Aldrich, St. Louis, USA). After incubation at 37 °C for 30 min, the fluorescence images were immediately taken using a fluorescence microscope (System Microscopy IX70; Olympus, Tokyo, Japan). For the detection of cellular superoxide, we used 5 μM MitoSOX (Invitrogen, Carlsbad, USA), and took images using excitation and emission filters of 543 nm and 565 nm, respectively. Fluorescent signals intensities of cells were counted using Image-Pro Plus (IPP) software.

## Lifespan experiments

Day 1 of adulthood was defined as t = 0, and the log-rank (Mantel-Cox) method was used to test the null hypothesis in Kaplan-Meier survival analysis, and evaluated using SPSS software. All experiments were carried out at 20°C for *C. elegans* and 25°C for *Drosophila*.

## Superoxide levels in *C. elegans*

To measure ROS levels, 100 4-day old adult worms, grown as described for the survival assays, were transferred to Eppendorf tubes containing 1.5 ml of M9 buffer, washed once in M9, and then stained for 30 min in 500 μl of M9 containing 5 μM MitoSOX red (Life Sciences/Invitrogen) while rotating in the dark. Worms were then washed once with M9, and transferred to 1.5% agarose pads on glass slides, covered, and immediately imaged within washing out the Mitosox red. Imaging was performed on using a fluorescence microscope (System Microscopy IX70; Olympus, Tokyo, Japan). At least 15 images were taken per genotype per replicate. Images were analysed in ImageJ, where average intensity of the head region was scored.

**mRNA isolation, reverse transcription and qPCR.**

Animals were collected and total RNA extracted using Trizol. mRNA quantification by reverse transcription and qPCR was performed as previously described (Chung *et al.* 2010). Samples were normalized to RP49 transcript levels. Experiments were conducted in biological duplicate. Primers used were published in previous reports, primers of *Drosophiladmsra*and *rp49* (Chung *et al.* 2010); primers of *C elegansmsra-1* and *act-4* (Minniti et al. 2009).

**MDA and cell viability assay**

The assay for MDA content was performed according to the protocols of the MDA kit (Jiangcheng bioengineering institute, Nanjing, China). And the cell survival rate tests were performed with WST-1 cell proliferation and cytotoxicity assay kit (Beyotime, Hangzhou, China) according to the manufacturer's protocol.

**HPLC assay method**

The HPLC assay method of DMSO was a modification of that described by Carpenter et al. The mobile phase contained 10% methanol (vol/vol) in high purity deionized water (>18 MΩ resistance), 20 μl of each sample was injected. The flow rate was 1.0 ml/min, and the UV detector was set at 214 nm. The column used was a Spherisorb OSD2 $C_{18}$ (5 μm) 4.6 × 250 mm. The cultured cells in 6-well plates were harvested after treatment, and then the samples were centrifuged for 5 min (×5,000 g). Supernatant was removed and cells were collected with 50 μl medium. Then the sample was sonicated for 1 min at 4 °C. The supernatant were diluted in mobile phase (1:10) and centrifuged for 10 min (×10,000 g) before injection. The HPLC device employed was a Shimadzu system. Data were acquired and analyzed with LC Solution.

**Worm survival rate under $H_2O_2$ induced oxidative stress**

We used 5-day-old adult worms were transferred to a 96-well plates (with 1–2 worms/well) containing 40 ml S-basal buffer containing hydrogen peroxide (10 mM)

at room temperature for 3 hours. Animals were tapped every 20-30 min and scored as dead when they did not respond to the platinum wire pick.

**MsrA RNAi nematode lifespan assay**

These experiments were performed using standard RNAi feeding protocols and the HT115 *E. coli* strain from the Ahringer RNAi feeding library (F43E2.5 clone). The exposure of worms to the*msra-1*RNAi clone was done from the embryo stage. Verification of interference was performed for every assay using RT-qPCR assay.

References:


Bowers K, Chow E, Xu H, Dror R, Eastwood M, Gregersen B, Klepeis J, Kolossvary I, Moraes M, Sacerdoti F, Salmon J, Shan Y, Shaw D (2006). Scalable Algorithms for Molecular Dynamics Simulations on Commodity Clusters. 43-43.

Case DA, Darden, T. A., III, T. E. C., Simmerling, C. L., Wang, J., Duke, R. E., Luo, R., Walker, R. C., Zhang, W., Merz, K. M., Roberts, B., Hayik, S., Roitberg, A., Seabra, G., Swails, J., Goetz, A. W., Kolossváry, I., Wong, K. F., Paesani, F., Vanicek, J., Wolf, R. M., Liu, J., Wu, X., Brozell, S. R., Steinbrecher, T., Gohlke, H., Cai, Q., Ye, X., Wang, J., Hsieh, M. J., Cui, G., Roe, D. R., Mathews, D. H., Seetin, M. G., Salomon-Ferrer, R., Sagui, C., Babin, V., Luchko, T., Gusarov, S., Kovalenko, A., and Kollman, P. A. (2012a). AMBER 12. *University of California: San Francisco*.

Case DA, Darden, T. A., III, T. E. C., Simmerling, C. L., Wang, J., Duke, R. E., Luo, R., Walker, R. C., Zhang, W., Merz, K. M., Roberts, B., Hayik, S., Roitberg, A., Seabra, G., Swails, J., Goetz, A. W., Kolossváry, I., Wong, K. F., Paesani, F., Vanicek, J., Wolf, R. M., Liu, J., Wu, X., Brozell, S. R., Steinbrecher, T., Gohlke, H., Cai, Q., Ye, X., Wang, J., Hsieh, M. J., Cui, G., Roe, D. R., Mathews, D. H., Seetin, M. G., Salomon-Ferrer, R., Sagui, C., Babin, V., Luchko, T., Gusarov, S., Kovalenko, A., and Kollman, P. A. (2012b). AMBER. *University of California, San Francisco*.

Chung H, Kim AK, Jung SA, Kim SW, Yu K, Lee JH (2010). The Drosophila

homolog of methionine sulfoxide reductase A extends lifespan and increases nuclear localization of FOXO. *FEBS Lett*. **584**, 3609-3614.

Lindorff-Larsen K, Piana S, Palmo K, Maragakis P, Klepeis JL, Dror RO, Shaw DE (2010). Improved side-chain torsion potentials for the Amber ff99SB protein force field. *Proteins*. **78**, 1950-1958.

Minniti AN, Cataldo R, Trigo C, Vasquez L, Mujica P, Leighton F, Inestrosa NC, Aldunate R (2009). Methionine sulfoxide reductase A expression is regulated by the DAF-16/FOXO pathway in Caenorhabditis elegans. *Aging Cell*. **8**, 690-705.

Neese F (2010). Orca–An Ab Initio, Density Functional and Semiempirical Program Package, Version 2.8. *University of Bonn, Bonn, Germany*.

Ranaivoson FM, Antoine M, Kauffmann B, Boschi-Muller S, Aubry A, Branlant G, Favier F (2008). A structural analysis of the catalytic mechanism of methionine sulfoxide reductase A from Neisseria meningitidis. *J Mol Biol*. **377**, 268-280.

Sali A, Blundell TL (1993). Comparative protein modelling by satisfaction of spatial restraints. *J Mol Biol*. **234**, 779-815.

Zhang ZX, Hempel PS, Han YL, Tjosvold D (2007). Transactive memory system links work team characteristics and performance. *J Appl Psychol*. **92**, 1722-1730.

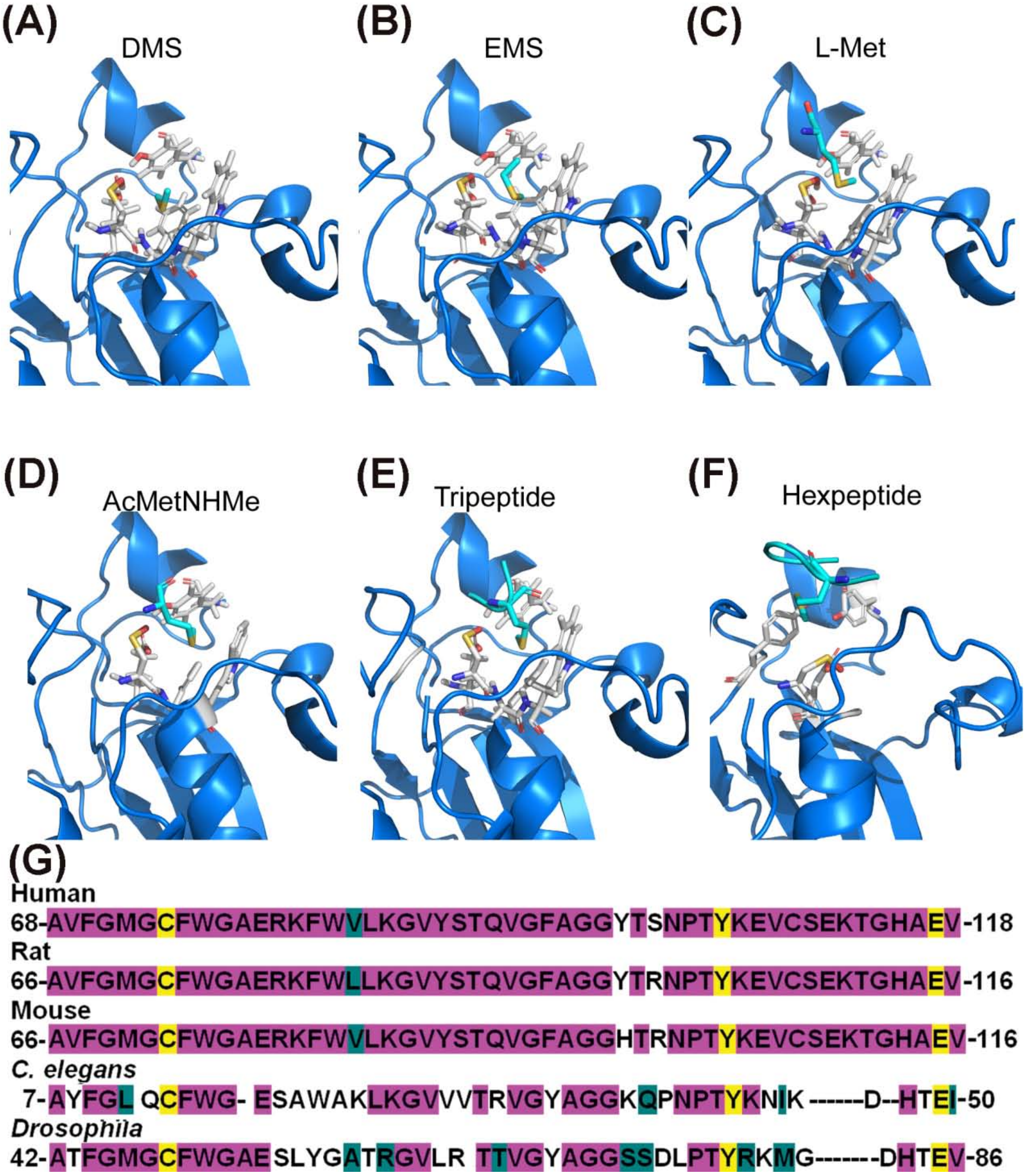


**Fig. S1** DMS serves as a dominant substrate of MsrA. The different reaction orientations of MsrA substrates were shown in **(A-F)**.DMS had similar orientations with L-Met. And L-Met had a better orientation than its derivative AcMetNHMe. Small peptides are set to mimic the reaction environment of protein Met. **(G)**The MsrA protein sequences of human, rat, *C. elegans* and *Drosophila* were aligned. The same residues were shown in purple, while similar residues were shown in green. The C72, Y103 and E115 were conservative and shown in yellow.

Table S1. Distances (Å) between atoms CYO-S/CYO-O and S atom; S from DMS, EMS, MPS, L-Met, AMN, Tripep, Hexpep

| | DMS-S | EMS[1]-S | MPS[2]-S | L-Met-S | AMN[3]-S | Tripeptide[4]-S | Hexpeptide[5]-S |
|---|---|---|---|---|---|---|---|
| CYO-S | 4.4 | 4.2 | 4.0 | 3.5 | 4.5 | 4.5 | 4.9 |
| CYO-O | 3.8 | 4.1 | 3.6 | 3.7 | 3.9 | 3.5 | 6.4 |

1: EMS, ethyl methyl sulfide

2: MPS, methyl propyl sulfide

3: AMN, AcMetNHMe, N-methyl acetyl methionine

4:Gly-Met-Gly

5: Pro-Met-Ala-Ile-Lys-Lys

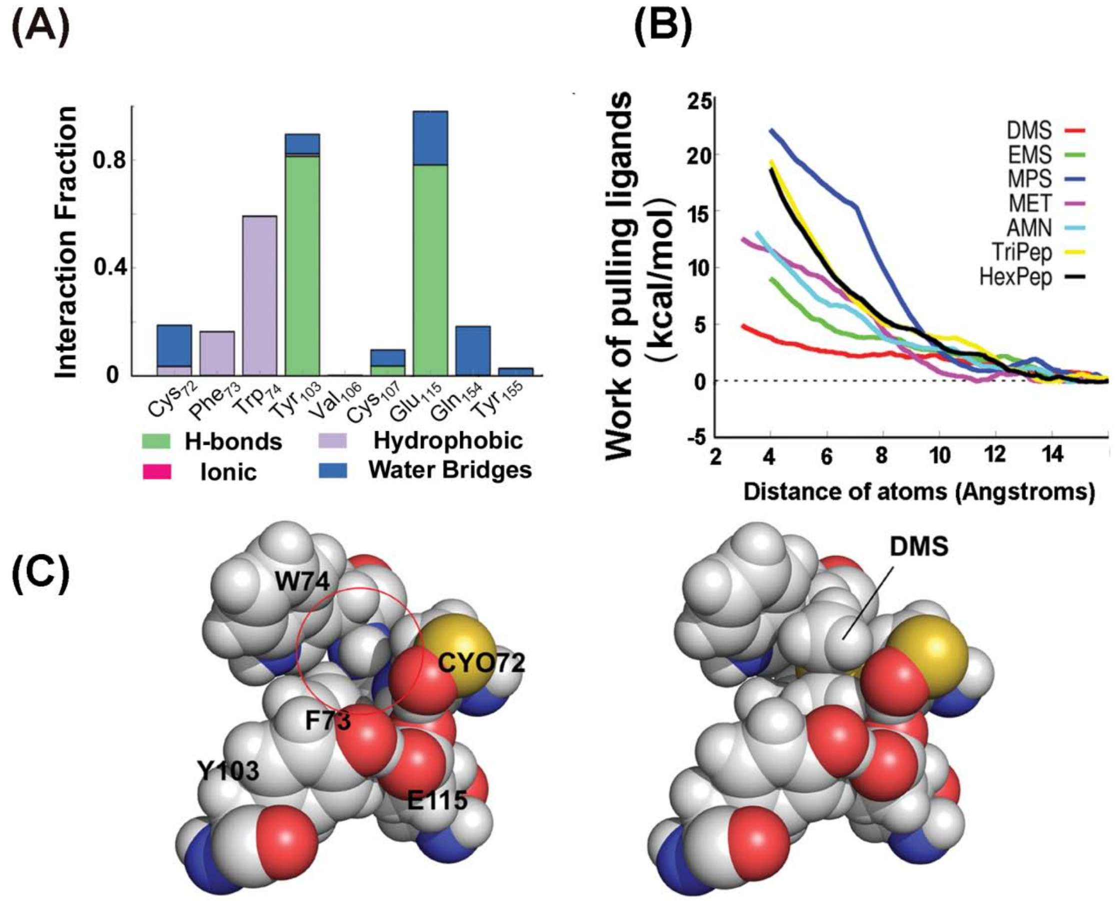


**Fig. S2** The catalytic pocket model of DMS-MsrA interaction. **(A)** The interaction between DMS and MsrA during simulation was shown in histogram. MsrA kept interactions with its substrate DMS, monitored throughout the whole simulation process. The stacked bar charts were normalized over the course of the trajectory, the value of y axis suggested the percentage of simulation time which the specific interaction is maintained. **(B)** Alignment of all refined complex. All of tasks were done with tug strength of 1.075kcal/(mol·Å$^2$), and the S-S distances were near equilibrium points as close as possible. Curves of works are indicated by different color. **(C)** A catalytic pocket was formed by the CYO72, F73, W74, Y103 and E115. When the binding site was occupied by DMS, F73 and W74 provided hydrophobic interactions with DMS to stable the complex.

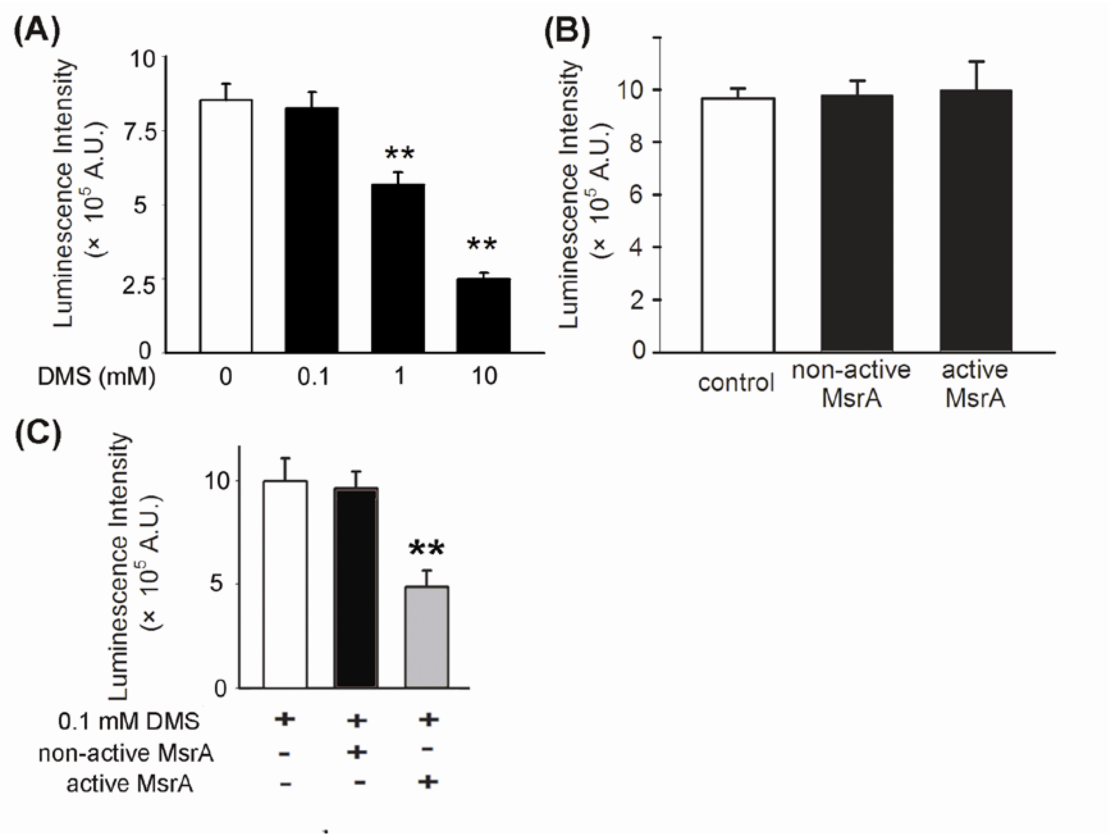


**Fig. S3** DMS decreases the luminal chemiluminescence *in vitro*, while MsrA enhances the OH· scavenge property. **(A)** The effect of 0.1, 1, 10 mM DMS on the luminescence signal intensity (n = 5, Student's t test, ** *P*< 0.01 vs. control). **(B)** The effect of MsrA or non-active MsrA alone on luminol-enhanced chemiluminescence was assessed (n = 4). **(C)** Inhibitory effect of 0.1 mM DMS on luminol-enhanced chemiluminescence in the presence of MsrA or non-active MsrA (1 μM) was determined (n = 5, Student's t test, ***P*< 0.01 vs. 0.1 mM DMS without DMS group).

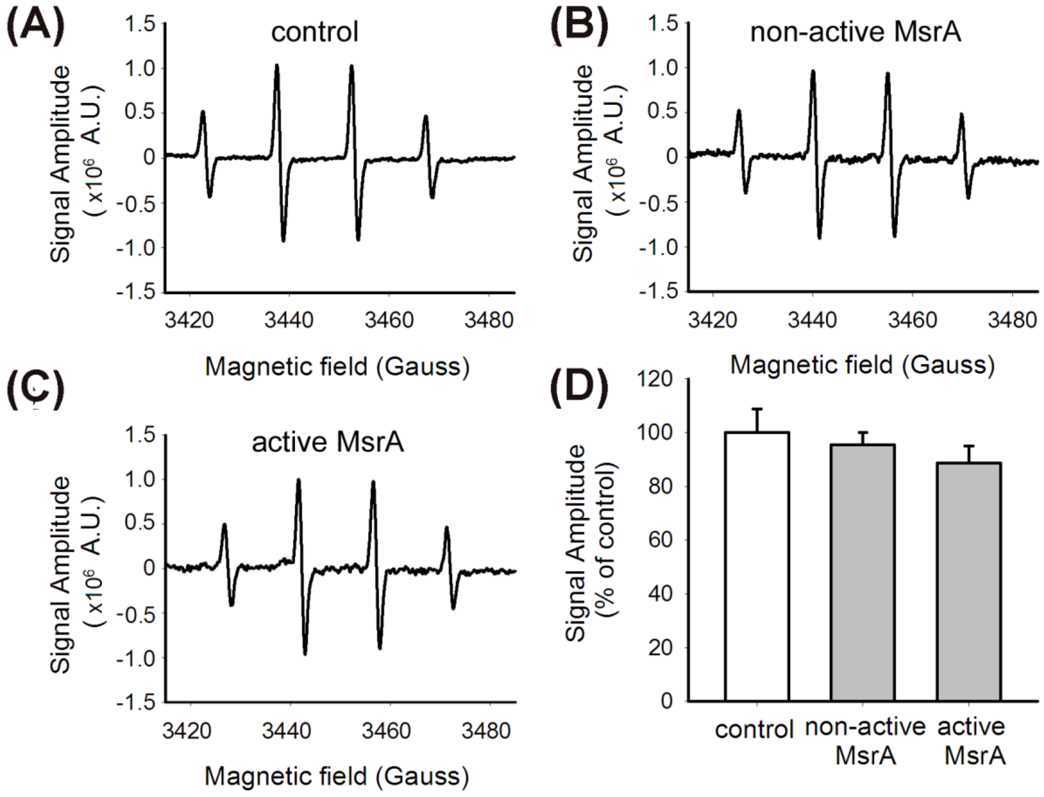


**Fig. S4** Neither MsrA nor non-active MsrA alone has a perceptible effect on ESR signals. **(A-C)** ESR spectra of the spin adduct of OH• radical observed during the reaction of 0.5 mM $H_2O_2$ containing 0.1 mM DMPO and 0.2 mM $FeSO_4$ with MsrA or non-active MsrA (n = 5). **(A)** Control, containing $H_2O_2$, $FeSO_4$ and PBS; **(B)** In the presence of non-active MsrA (3 μg); **(C)** In the presence of MsrA (3 μg); **(D)** Levels of ESR peak intensity were expressed as a relative change in comparison with the untreated control, which was set to 100%.

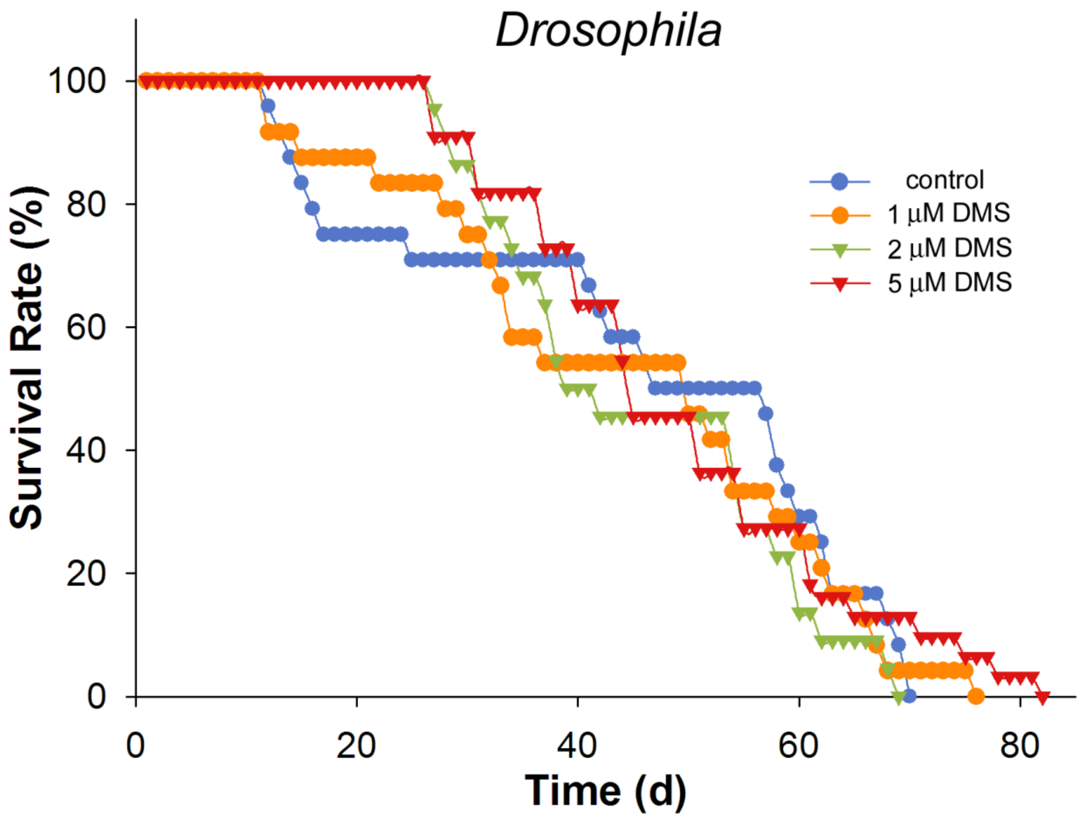


**Fig. S5** DMS does not prolong the lifespan of *Drosophila* in low concentrations. The lifespan analyses of wild type flies exposed to DMS (1, 2, or 5 μM) were shown in plot graph (n = 100 from 3 independent experiments, $P > 0.05$ vs. control).

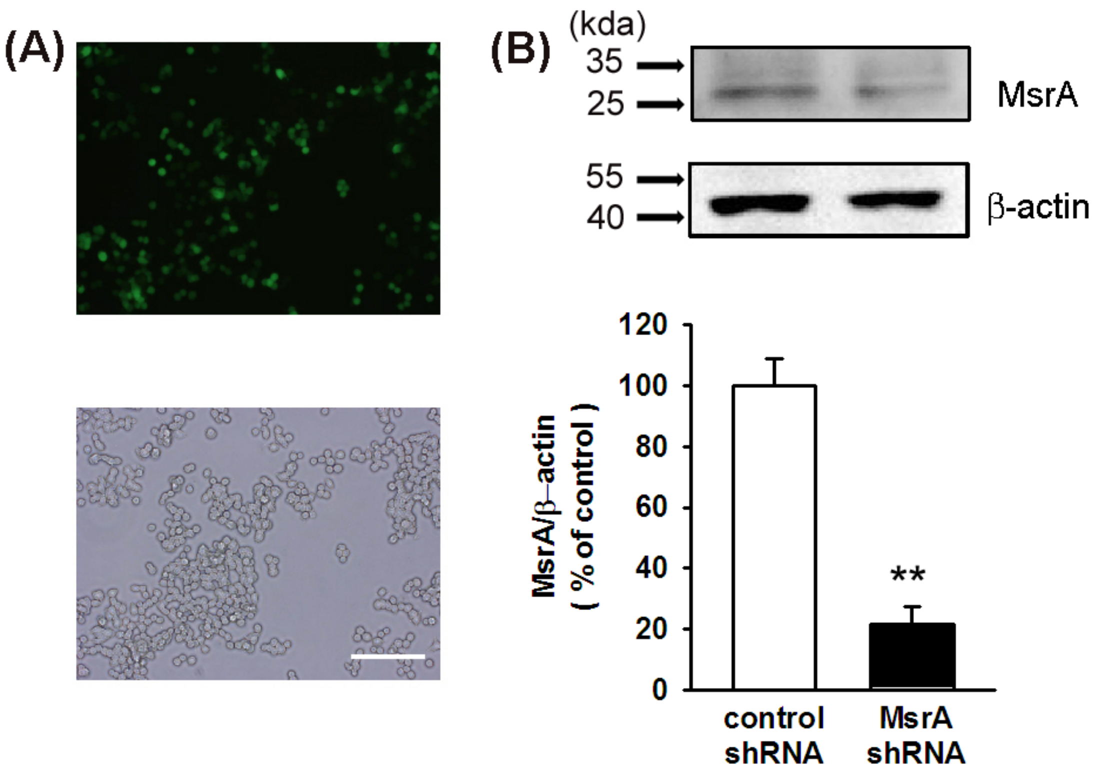


**Fig. S6** MsrA shRNA significantly inhibits the expression of MsrA in PC12 cells. **(A)** Transfected cells were observed to confirm more than 80% of cells were GFP-positive. The scale bar represents 100 μm. **(B)** The expression of MsrA was detected and expressed as a relative change in comparison with the control shRNA treatment (n=6, ***P* < 0.01, Student's t-test).

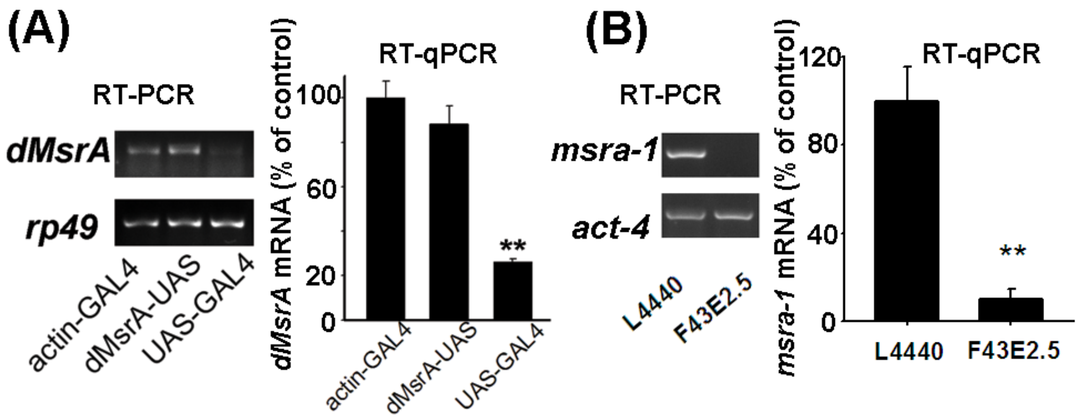


**Fig. S7** The effects of MsrA RNAi are evaluated by RT-PCR and RT-qPCR. **(A)**The representative figure of RT-PCR was showed. And the *dMsrA* mRNA levels of different fly strains were quantitated by real-time qPCR. (n = 4, ***P* < 0.01 vs. actin-GAL4 group, Student's t test). **(B)**The representative figure of RT-PCR was showed. And the *msra-1* mRNA levels of different worm strains were quantitated by real-time qPCR. (n = 4, ***P* < 0.01 vs. L4440 group, Student's t test).

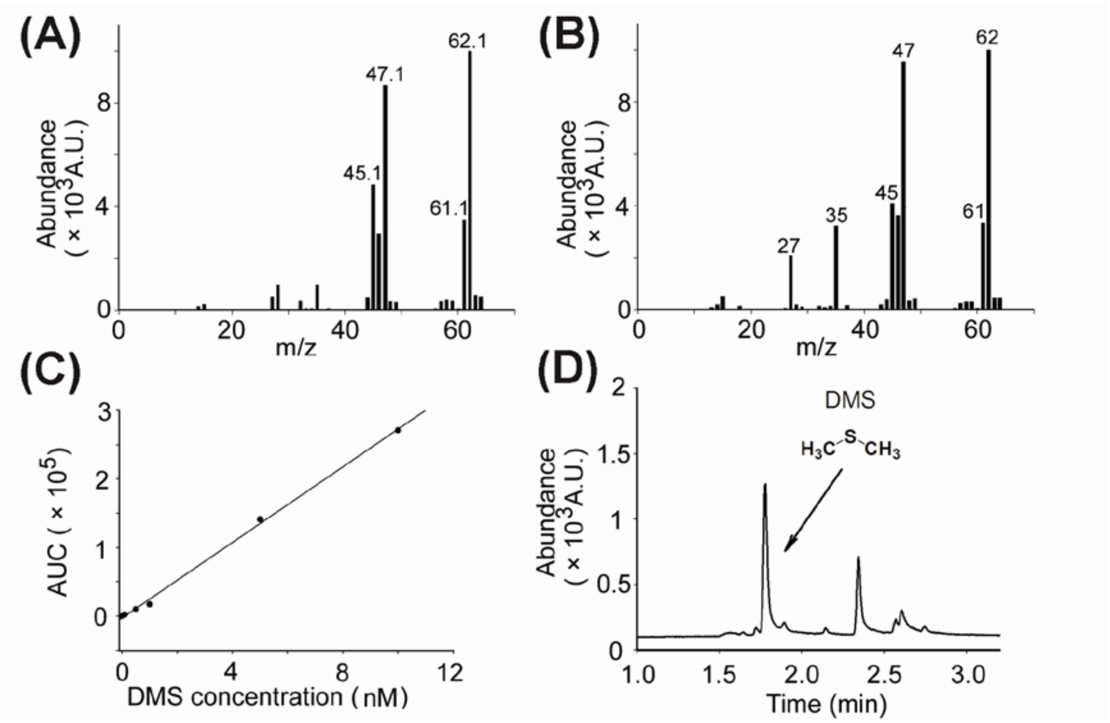


**Fig. S8** The detection method of DMS is built using GC-MS. **(A)** Mass spactrum of tested sample. **(B)** Ion fragment of DMS in database. **(C)** Standard curve was built to quantitate DMS concentration. **(D)** Representative GC-MS spectrum of DMS was showed.
